# A Vanished Association Between Proton Pump Inhibitors and Clostridioides Difficile Infection After Minimizing Bias

**DOI:** 10.3390/jcm15010230

**Published:** 2025-12-27

**Authors:** Bin Wu, Zhiyao He, Ting Xu

**Affiliations:** 1 Department of Pharmacy, West China Hospital, Sichuan University, Chengdu 610041, China; binw83@hotmail.com (B.W.); zhiyaohe@scu.edu.cn (Z.H.); 2 West China School of Pharmacy, Sichuan University, Chengdu 610041, China

**Keywords:** proton-pump inhibitors, Clostridioides difficile infection, disproportionality analysis

## Abstract

**Background:** The gut microbiome might be affected by proton-pump inhibitors (PPIs), increasing the risk of Clostridioides difficile infection (CDI); however, the association between PPIs and Clostridioides difficile infection (CDI) remains controversial. **Aim**: The aim of this study is to reevaluate the association between PPIs and CDI based on pharmacovigilance data, taking competition bias into account. **Methods:** PPI-related CDI adverse event reports, based on the Food and Drug Administration adverse event reporting system database from 2004 to 2023, were analyzed. Included PPI cases were stratified into CDI and non-CDI groups. Disproportionality analysis was performed using the reporting odds ratio (ROR) and information component (IC). The effect of competition bias on signal detection was quantitatively investigated. Age-stratified analyses were conducted to assess residual confounding. **Results:** A total of 238,470 PPI reports were included, with 1268 cases in the CDI group and 237,202 cases in the non-CDI group. Initial analysis revealed a significant PPI-CDI association (ROR = 2.36, 95% confidence interval (95%CI) 2.19 to 2.53; IC = 1.21, 95%CI 0.97 to 1.45), with CDI signals detected for five PPI agents, including pantoprazole, omeprazole, lansoprazole, rabeprazole, and dexlansoprazole. After excluding competition from antibacterial drugs, CDI signal strength decreased substantially (ROR = 1.47, 95%CI 1.34 to 1.62; IC = 0.55, 95%CI 0.23 to 0.87), retaining a significant CDI signal only for rabeprazole and pantoprazole. Upon further exclusion of antibacterial or immunosuppressive drug users and renal injury event cases, CDI signal strength decreased (ROR = 1.48, 95%CI 1.32 to 1.66; IC = 0.56, 95%CI 0.18 to 0.94), with pantoprazole as the sole CDI signal drug. Age-stratified analyses demonstrated complete signal loss after antibacterial drug adjustment across all age groups. **Conclusions:** The current large-scale pharmacovigilance study indicated that the observed PPI-CDI association may be mediated predominantly by antibacterial drug co-exposure rather than PPI direct causation.

## 1. Introduction

Well-known proton-pump inhibitors (PPIs), including omeprazole, pantoprazole, lansoprazole, rabeprazole, esomeprazole, dexlansoprazole, and dexrabeprazole, are a group of agents that work by inhibiting gastric acid secretion [1]. PPIs have been used widely to treat gastroesophageal reflux disease, peptic ulcer disease, and Helicobacter pylori infection or to prevent the side effects caused by other drugs such as corticosteroids, nonsteroidal anti-inflammatory drugs, chemotherapy, or anticoagulants [2]. As drug research has advanced, some novel drugs, including vonoprazan and tegoprazan, have been added to the PPI family [3].

PPIs are widely used in hospital settings, in the community, and even at home. Outside of use in indicated cases, PPIs continue to be overused in cases of off-label prescribing in excessive dosages and for unnecessary long-term treatment [4,5]. As their use has become widespread, the safety issues associated with PPIs have increasingly drawn the attention of patients, clinicians, and researchers [6]. The primary known side effects of PPIs are abdominal pain and diarrhea, attributed to the inhibition of gastric acid secretion. Studies on the long-term use of PPIs may also note some other potential adverse effects, including renal injury, fractures, hypergastrinemia, dementia, and Clostridioides difficile infection (CDI) [7]. For instance, the immune response in the tubule-interstitium might be induced by PPIs and their metabolites, causing interstitial inflammatory infiltrate and acute interstitial nephritis, resulting in various types of renal injury [8]. Hypochlorhydria and hypergastrinemia can also be caused by PPIs, leading to more insoluble calcium and less calcium absorption, increasing fracture risk [9]. β- and γ-secretase metabolism, vitamin B12 deficiency, and choline acetyl transferase inhibition can be induced by PPIs, increasing the risk of dementia [10].

CDI is a significant threat to public health and is a challenge in clinical practice. A meta-analysis that included 229 publications from 41 countries estimated the cumulative global incidence of CDI to be 41.94 cases per 100,000 population per year, and an even higher rate of healthcare facility-associated CDI at 2.24 per 1000 admissions per year [11]. In 2012, CDI-associated diarrhea that was related to PPI treatment was announced by a Food and Drug Administration (FDA) Drug Safety communication. A myriad of observational studies were conducted, indicating that patients who had received PPIs suffered an increased risk of CDI-related events. More than 10 systematic reviews were undertaken to investigate PPI therapy and the risk of CDI [12,13,14,15,16,17,18,19,20,21,22,23]. However, the relationship between PPIs and CDI remained uncertain. Some meta-analyses of these observational studies showed consistencies and even found that this association between PPIs and CDI-related events was amplified [16,19,24]. The hypothesis of the causal relationship between PPIs and CDI was mainly based on the effects PPIs have on the diversity of the gut microbiome [25]. Other meta-analysis found evidence supporting a cause–effect relationship between PPIs and CDI to be of very low quality [22]. The primary challenge to the association between PPIs and CDI was unmanageable confounders, such as age, gender, primary disease, hospitalization, and exposure to competing drugs [26]. Therefore, it was necessary to conduct a comprehensive analysis of the existing studies on this hot topic.

Systemic antibacterial drugs and immunosuppressive drugs are the top two classes of confounding agents in terms of studying the relationship between PPIs and CDI. Studies have demonstrated significant associations between CDI and systemic antibiotic administration [27,28], as well as immunosuppressive therapy [29]. The combined use of systemic antibacterial drugs and PPIs could increase the risk of CDI [30]. Randomized controlled trials failed to reveal the relationship between PPIs and CDI events, which were often set as secondary outcome measurements [26]. Observational studies showed inconsistent results for an association between PPIs and CDI, despite attempts to control for confounding factors [31]. A recent pharmacovigilance study investigating the association between CDI and both vonoprazan and conventional PPIs failed to adjust for critical confounders, particularly the concurrent use of antibacterial drugs [32].

The FDA’s adverse event reporting system (FAERS) is a commonly used source in pharmacovigilance analysis [33]. The spontaneous reporting of adverse event data by FAERS could be used to analyze the association between specific drugs and target adverse events [34]. We previously conducted a disproportionality analysis of PPIs and dementia events considering competition bias using FAERS data [10]. That study revealed that more than 20% of PPI cases reported renal injury events [10], which could induce an event-related competition effect [35], masking the analysis of other PPI-related adverse events. Therefore, it was necessary to perform the current pharmacovigilance research on PPIs and CDI events, specifically to evaluate the influence of competition bias.

The aim of the present study is to investigate the association between PPI treatment and CDI events using disproportionality analysis with quantitative adjustment for competition bias, including combination treatment with antibacterial drugs or immunosuppressive drugs, or PPI-related renal injury event reports.

## 2. Materials and Methods

The current study comprises a retrospective disproportionality analysis aiming to investigate the association between PPI treatment and CDI events, with quantitative adjustment for competition bias, based on FAERS safety report cases between January 2004 and December 2023.

### 2.1. Data Source

The FAERS dataset consists of seven data tables including the DEMO, DRUG, REAC, OUTC, RPSR, THER, and INDI tables. The DEMO table is a patient demographic information table, the DRUG table is a drug information table, the REAC table is an adverse events information table, the OUTC table is a patient outcomes information table, the RPSR table is a report sources information table, the THER table is a drug therapy date information table, and the INDI table is a drug indication table [36].

### 2.2. Study Procedure

Original data from the FAERS Quarterly Data Extract Files website was captured and imported into a local Microsoft SQL server 2022 software (Microsoft Corporation, Redmond, WA, USA) setup. Duplicated reports were removed based on the CASEID of the DEMO table, and the “deleted” cases were further removed [37]. MedEx software (MedEx UIMA 1.3.8, Vanderbilt university, Nashville, TN, USA) was used to standardize the various drug names into a “generic name” [38].

Nine single-component PPIs, coded by the WHO Anatomical Therapeutic Chemical (ATC) classification system as A02BC, were identified as the target drugs in the current study. The nine PPIs included were omeprazole (A02BC01), pantoprazole (A02BC02), lansoprazole (A02BC03), rabeprazole (A02BC04), esomeprazole (A02BC05), dexlansoprazole (A02BC06), dexrabeprazole (A02BC07), vonoprazan (A02BC08), and tegoprazan (A02BC09). PPIs reported as primary suspect (PS) or secondary suspect (SS) drugs were included in the current study. Stratified analysis was performed based on the drug’s suspect role or different ages for sensitivity analysis.

Adverse events in the REAC table and indications in the INDI table were both coded by the Medical Dictionary for Regularly Activities (MedDRA). The coded terms used were designated as preferred terms (PTs) [39]. CDI events were identified in the REAC table using 9 PTs (Supplementary Table S1) [40].

To analyze the competition bias regarding CDI signal detection among PPI cases, co-therapy drugs including immunosuppressive drugs and systemic antibacterial drugs were chosen as the competing drugs; moreover, the renal injury adverse event was chosen as the competing event. Immunosuppressive drugs were identified by the ATC code L04 (Supplementary Table S2), and systemic antibacterial drugs by the ATC code J01 (Supplementary Table S3). Renal injury cases were identified based on the MedDRA High-Level Group Term (HLGT) search (Supplementary Table S4).

All the primary and secondary suspect PPI cases, except cases in which the drug was prescribed for CDI, were included in the current study.

### 2.3. Statistical Analysis

Two disproportionality analysis (DPA) algorithms, including a frequency method (reporting odds ratio, ROR) and a Bayesian method (information component, IC) (Supplementary Table S5), were employed to investigate the association between PPIs and CDI. The background data for the DPA algorithms were all FAERS cases except the PPI cases. In the present study, the ROR was the degree of disproportionate reporting of CDI events for target PPIs compared with the background data; a bigger ROR value indicated a stronger association between PPIs and CDI. A CDI signal was detected when the reported case number ≥ 3, and the lower limit was a 95% confidence interval (95%CI) of a ROR > 1, and the lower limit of a 95%CI for IC > 0. A CDI signal indicated a significant association between a CDI event and PPI treatment.

The effect of competing drugs and competing events on the detection of the CDI signal was investigated. First, the ROR, IC, and their 95%CI values among all included cases were calculated, then the same parameter values were recalculated once all PPI cases with competing drugs or competing events were excluded. The CDI signals before and after the exclusion of competition factors were compared to detect the effect of competition bias.

CDI signals in different age groups, including the 18 years and below, 18 to 64 years, and 65 years and above groups, were further analyzed.

### 2.4. Statistical Software

The statistical analyses were conducted using Microsoft Excel version 2021 (Microsoft corporation, Redmond, Washington, DC, USA) and SPSS version 25.0 (IBM corporation, Armonk, NY, USA).

## 3. Results

### 3.1. Identification of PPI Cases in FAERS

**Figure 1:**
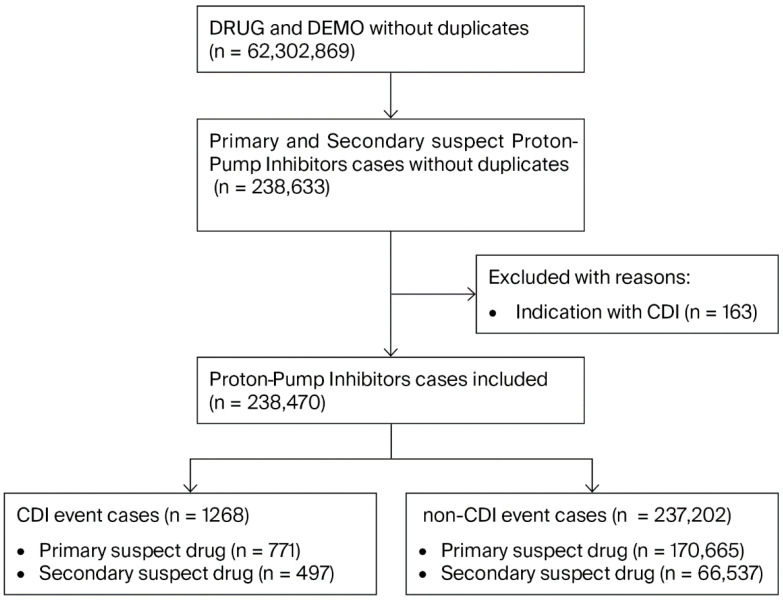
Flow chart of identifying CDI cases out of proton-pump inhibitor cases from FAERS database. DRUG: the drug table; DEMO: the demographic table; CDI: Clostridioides difficile infection.

A total of 238,470 cases with PPIs reported as primary or secondary suspect drugs were finally included from FAERS (Figure 1). In total, 1268 cases (771 primary suspect PPI cases and 497 secondary suspect PPI cases) were reported with CDI events, and 237,202 cases were reported without CDI events.

### 3.2. Characteristics of PPI Cases Reported in FAERS

The characteristics of the 238,470 PPI cases are shown in Table 1. The top three primary suspect PPIs with a CDI event were pantoprazole (245 cases), omeprazole (193 cases), and esomeprazole (156 cases). A total of 22,763 primary suspect PPI cases were reported with the combined use of antibacterial drugs, and 6372 with the combined use of immunosuppressive drugs. The reported CDI proportions were similar between the female (0.45%) and male (0.42%) groups. The highest CDI proportion (0.75%) was in the 65 years and above age group. The highest CDI proportion (0.87%) was reported by healthcare professionals. North America reported more (71.95%) of the primary suspect PPI cases; however, the highest CDI proportion (0.89%) was reported in Europe.

The annual reports of PPI cases from 2004 to 2023 revealed a sharp increase in 2019, as shown in Supplementary Figure S1, with a rapid rise in PPI-related renal injury cases. The trend of CDI cases associated with PPI treatment is shown in Supplementary Figure S2.

### 3.3. Disproportionality Analysis Based on All PPI Cases

CDI signals based on all primary suspect PPIs were analyzed, and a significant association between PPIs and CDI was detected (ROR = 2.36, 95%CI 2.19 to 2.53; IC = 1.21, 95%CI 0.97 to 1.45). Pantoprazole, rabeprazole, omeprazole, and lansoprazole all showed CDI signals (Table 2).

CDI signals based on all primary and secondary suspect PPIs were further analyzed, and significant associations were detected between PPIs and CDI (ROR = 2.82, 95%CI 2.67 to 2.98; IC = 1.46, 95%CI 1.27 to 1.64). Pantoprazole, omeprazole, lansoprazole, rabeprazole, and dexlansoprazole all showed CDI signals (Supplementary Table S6).

### 3.4. Disproportionality Analysis After ISD Cases Excluded

Cases where PPIs were combined with immunosuppressive drugs (6372 cases, 3.72%) were excluded from all primary suspect PPIs, and the strength of the CDI signal decreased slightly (ROR = 2.31, 95%CI 2.15 to 2.49; IC = 1.19, 95%CI 0.94 to 1.43). Pantoprazole, rabeprazole, omeprazole, and lansoprazole all showed a CDI signal (Table 2).

Cases where PPIs were combined with immunosuppressive drugs (18,611 cases, 7.80%) were further excluded from all primary and secondary suspect PPIs, and the strength of the CDI signal decreased slightly (ROR = 2.74, 95%CI 2.58 to 2.91; IC = 1.42, 95%CI 1.22 to 1.61). Omeprazole, pantoprazole, lansoprazole, and rabeprazole all showed a CDI signal (Supplementary Table S6).

### 3.5. Disproportionality Analysis After ABD Cases Excluded

Cases where PPIs were combined with antibacterial drugs (22,763 cases, 13.28%) were excluded from all primary suspect PPIs, and the strength of the CDI signal decreased significantly (ROR = 1.47, 95%CI 1.34 to 1.62; IC = 0.55, 95%CI 0.23 to 0.87). Only rabeprazole and pantoprazole showed a CDI signal (Table 2).

Cases where PPIs were combined with antibacterial drugs (40,823 cases, 17.12%) were further excluded from all primary and secondary suspect PPIs, and the strength of the CDI signal decreased significantly (ROR = 1.51, 95%CI 1.39 to 1.64; IC = 0.58, 95%CI 0.3 to 0.85). Only pantoprazole and omeprazole showed a CDI signal (Supplementary Table S6).

### 3.6. Disproportionality Analysis After ABD or ISD Cases Excluded

Cases where PPIs were combined with either immunosuppressive drugs or antibacterial drugs (27,140 cases, 15.83%) were excluded from all primary suspect PPIs, and the strength of the CDI signal further decreased (ROR = 1.40, 95%CI 1.27 to 1.55; IC = 0.48, 95%CI 0.15 to 0.81). Only rabeprazole and pantoprazole showed a CDI signal (Table 2).

Cases where PPIs were combined with either immunosuppressive drugs or antibacterial drugs (53,070 cases, 22.25%) were excluded from all primary and secondary suspect PPIs, and the strength of the CDI signal further decreased (ROR = 1.30, 95%CI 1.19 to 1.43; IC = 0.37, 95%CI 0.07 to 0.68). Only pantoprazole showed a CDI signal (Supplementary Table S6).

### 3.7. Disproportionality Analysis After RI Cases Excluded

PPI cases in which a renal injury event was reported (56,663 cases, 33.05%) were excluded from all primary suspect PPI cases, and the strength of the CDI signal increased (ROR = 2.83, 95%CI 2.62 to 3.07; IC = 1.48, 95%CI 1.21 to 1.74). However, only pantoprazole, lansoprazole, and omeprazole showed a CDI signal (Table 2).

PPI cases in which a renal injury event was reported (62,874 cases, 26.37%) were excluded from all primary and secondary suspect PPI cases, and the strength of the CDI signal still increased (ROR = 3.23, 95%CI 3.04 to 3.44; IC = 1.65, 95%CI 1.45 to 1.85). Lansoprazole, pantoprazole, and omeprazole showed a CDI signal (Supplementary Table S6).

### 3.8. Disproportionality Analysis After ABD, ISD, or RI Cases Excluded

PPI cases combined with immunosuppressive drugs or antibacterial drugs and cases in which a renal injury event was reported (69,655 cases, 40.63%) were excluded from all primary suspect PPIs, and the strength of the CDI signal still decreased (ROR = 1.48, 95%CI 1.32 to 1.66; IC = 0.56, 95%CI 0.18 to 0.94). Only pantoprazole showed a CDI signal (Table 2).

PPI cases combined with immunosuppressive drugs or antibacterial drugs, and cases in which a renal injury event was reported (98,894 cases, 41.47%) were excluded from all primary and secondary suspect PPIs, and the strength of the CDI signal still decreased (ROR = 1.34, 95%CI 1.2 to 1.48; IC = 0.41, 95%CI 0.07 to 0.76). Only pantoprazole showed a CDI signal (Supplementary Table S6).

### 3.9. Disproportionality Analysis Based on Different Age Groups

**Figure 2:**
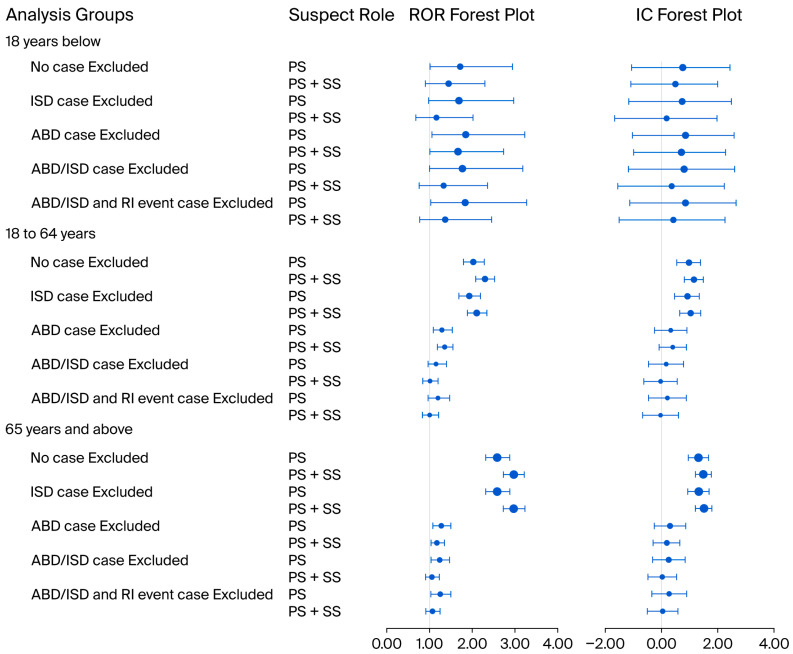
Disproportionality analysis of Clostridioides difficile infection signal based on different age groups. ROR: reporting odds ratio; 95%CI: 95% confidence interval; IC: information component; ISD: immunosuppressive drugs; ABD: antibacterial drugs; RI: renal injury; PS: primary suspect drug; PS+SS: primary or secondary suspect drug; the bubble size represents the CDI proportion.

CDI signals based on different age groups were analyzed, including 18 years and below, 18 to 64 years old, and 65 years and above groups. The primary suspect PPI cases were analyzed, and CDI signals were detected in the 18 to 64 years old, and 65 years and above groups in all PPI case groups. Once cases where PPIs were combined with antibacterial drugs were excluded, the CDI signal was lost among all three age groups (Figure 2). The primary and secondary suspect PPI cases were further analyzed, and a consistent conclusion was discovered in the primary suspect PPI analysis. The CDI signal of specific PPI agents is shown in Supplementary Tables S7 and S8.

### 3.10. Outcomes of CDI Cases in Different Age Groups

**Figure 3:**
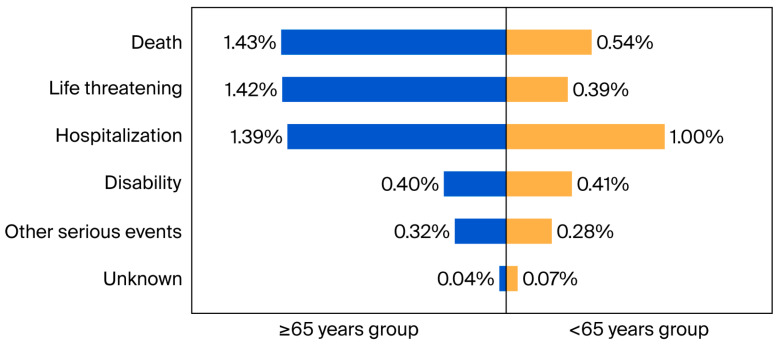
Outcomes of Clostridioides difficile infection related to proton-pump inhibitor treatment for different age groups.

Finally, the seriousness of the outcomes of CDI cases related to PPI treatment between two age groups were analyzed (Figure 3). The proportion of outcomes, including death (1.43% vs. 0.54%), life threatening (1.42% vs. 0.39%), hospitalization (1.39% vs. 1.00%), and other serious events (0.32% vs. 0.28%), were higher in the 65 years and above group compared with the 65 years and below group.

## 4. Discussion

In the current study, a disproportionality analysis was performed to investigate the association between PPIs and CDI events while rigorously adjusting for confounding factors including competing drugs and competing events. Our analysis demonstrates that, after rigorously adjusting for competition bias, particularly concomitant antimicrobial drug use, the previously reported association between PPIs and CDI was attenuated to non-significance, indicating that the observed PPI-CDI association may be predominantly mediated by antibacterial drug co-exposure rather than direct causation by PPIs.

The relationship between PPI use and the risk of CDI has always been a pressing issue. Studies focused on PPI use and CDI risk did not reach consistent conclusions. At least 12 systematic reviews or meta-analysis have focused on the association between PPI treatment and the risk of CDI over a period that spans from 2012 to 2025 [12,13,14,15,16,17,19,20,21,22,23]. All 12 studies included cohort studies and case–control studies for data synthesis. Nine studies evaluated PPI therapy and the risk of CDI. Critically low- or low-quality evidence indicated that PPI treatment may be associated with an increased risk of developing CDI, with OR values varying from 1.39 to 2.34 [12,13,14,16,17,20,21,22,23]. Three studies evaluated PPI therapy and the risk of recurrent CDI. Critically low- or low-quality evidence indicated that PPIs may increase the risk of recurrent CDI, with OR values varying from 1.69 to 2.51 [15,18,19]. Additionally, one study evaluated the association between PPI dose or duration and the risk of CDI. Critically low-quality evidence indicated a possible increase in the risk of CDI with increasing dose (RR = 1.05, 95%CI (0.89, 1.23) per 10mg DDD) and duration (RR = 1.02, 95%CI (1.00, 1.05) per day) of PPI therapy [17].

Although most systematic reviews and meta-analysis reported on the influence of confounding factors, it was hard to conduct a thorough analysis of control for confounding factors due to the limitations of the original study designs. Therefore, it was essential to conduct a further study to analyze the relationship between PPIs and CDI, fully considering the influence of important sources of bias.

This disproportionality analysis, adjusting for confounding factors including competing drugs and competing events, serves as a timely supplement to the current research. The results of the current study challenge the conclusions of earlier observational studies that identified PPIs as an independent risk factor for CDI, with odds ratios ranging from 1.74 to 2.51 [12,18]. Our findings also challenge the conclusion of earlier pharmacovigilance studies which revealed significant associations between PPIs and CDI, with RORs (95%CIs) ranging from 1.42 (1.34 to 1.51) to 2.51 (1.92 to 3.28) based on FAERS and JADER, respectively [32]. These studies, however, often lacked granular adjustments for exposure to antibacterial drugs, a key confounder. Contrasting with these findings, recent studies on vonoprazan, a potent acid suppressant, revealed no additional CDI risk compared with conventional PPIs, despite its stronger acid-inhibiting effects [41,42].

Antibacterial drug use emerged as the most significant confounder in the present analysis. Our findings aligned with Moreels et al., who demonstrated that the co-exposure of PPIs and systemic antibacterial drugs synergistically increased CDI risk and recurrence [30]. This interaction underscores the importance of stratifying analyses by antibiotic exposure, as highlighted by Gordon et al., who found no significant difference in CDI incidence between patients on antibacterial drugs alone versus antibacterial drugs combined with PPIs [43]. Furthermore, the frequent co-prescription of PPIs with antibacterial drugs in clinical settings created bias, leading to inflated estimates in unadjusted models [44]. These insights emphasize the necessity of disentangling the effects of competing drugs in pharmacovigilance studies.

In addition to antibacterial drugs, this study also investigated the influence of other sources of competition bias on CDI signal detection. The co-administration of PPIs with immunosuppressive agents, another category of competing drugs, did not significantly affect CDI signal detection. Temporal trends analysis revealed a progressive increase in PPI-related studies indexed in PubMed, peaking in 2020. Similarly, the FAERS database exhibited a rise in PPI-related adverse event reports over time, with a notable surge in renal injury cases around 2019. Renal injury events accounted for over 20% of primary suspected PPI-related cases in FAERS. Therefore, when analyzing other adverse event signals for PPIs, renal injury might act as a competing event that could introduce masking bias. When competing event cases are excluded, the signals for other adverse events for the target drug tend to become more pronounced. This was consistent with our results when renal injury cases were excluded. Moreover, after eliminating the influence of competing events, the CDI signal weakened again when considering the impact of competitive drugs, which is consistent with the hypothesis of the current study.

The mechanism supporting PPI treatment increasing CDI risk includes the notion that PPIs inhibit the secretion of gastric acid, consequently inhibiting the activity of neutrophils and enhancing the expression of CDI toxins, leading to excessive bacterial growth and spore survival, a weak bactericidal effect of neutrophils and the increased pathogenicity of CDI [45]. However, whether and how PPIs cause the increased acquisition of CDI remains unknown. The current study’s results challenge the strength of this hypothesis, indicating that PPI treatment is more likely to be a confounder of antibiotic use. A diverse gut microbiota could prevent CDI, while the disruption of the microbiota could lead to a decrease in or even loss of this anti-infection effect, and the proliferation of CDI [46]. Antibiotic treatment is an important factor causing the disruption of the gut microbiota and leading to the risk of CDI increasing [47]. In addition, the specific types of antibiotic molecules, the duration of antibiotic treatment, and the patient’s status such as aged or suffering from obesity, hypoalbuminemia, impaired humoral immunity, or renal impairment, may be risk factors that are related to CDI [48]. Therefore, confounding factors including antibiotics cannot be ignored when studying the relationship between PPIs and the risk of CDI.

The outcomes of CDI event cases with PPI treatment revealed that adults aged 65 years and above experienced more severe CDI outcomes compared with the 65 years and below population. However, our data suggested that age-related physiological changes, rather than PPI use itself, drive this prognostic risk. Khanna et al. found that acid suppression did not independently predict CDI outcomes in elderly populations after adjusting for frailty and polypharmacy [31], which is consistent with our findings.

While the current study framework incorporated confounders, several limitations persist. Firstly, disproportionality analyses are inherently susceptible to reporting biases; therefore, although we had taken renal injury into account as a competing event, the underreporting of mild CDI cases or unmeasured reporting bias still exist. Secondly, the generalizability of the findings to non-hospitalized populations requires validation, as do CDI risk profiles that differ between community and healthcare settings [49]. Thirdly, missing data in the drug therapy date information table was particularly serious, so we did not include a time to onset analysis in the current study.

## 5. Conclusions

The disproportionality analysis added evidence that the association between PPIs and CDI may be mediated by confounding by antibacterial drug co-treatment, rather than being the result of direct causation by PPIs. Although further high-quality prospective studies are still needed, these findings may call for a paradigm shift in considerations in clinical practice, prioritizing antimicrobial stewardship over PPI restriction in CDI prevention strategies. We recommend conducting high-quality prospective real-world studies to investigate the impact of the combined use of antibiotics, including specific types of antibiotic molecules or durations of antibiotic treatment, on the relationship between PPI treatment and CDI risk.

## Figures and Tables

**Table 1:** Characteristics of included proton-pump inhibitor cases reported in FAERS database.

Characteristics	CDI Event Cases				Non-CDI Event Cases			
	PS PPI Cases		SS PPI Cases		PS PPI Cases		SS PPI Cases	
	Number/n	Proportion/%	Number/n	Proportion/%	Number/n	Proportion/%	Number/n	Proportion/%
PPIs	771		497		170,665		66,537	
Pantoprazole	245	0.83	155	0.75	29,292	99.17	20,611	99.25
Omeprazole	193	0.51	171	0.91	37,340	99.49	18,562	99.09
Esomeprazole	156	0.23	50	0.35	67,969	99.77	14,273	99.65
Lansoprazole	124	0.47	104	1.10	26,510	99.53	9370	98.90
Dexlansoprazole	26	0.46	5	0.57	5686	99.54	869	99.43
Rabeprazole	26	0.68	8	0.31	3816	99.32	2533	99.69
Vonoprazan	1	1.89	4	1.24	52	98.11	318	98.76
Tegoprazan	0	0.00	0	0.00	0	0.00	1	100.00
PPI combined with other drugs								
Antibacterial drugs	349	1.53	346	1.92	22,414	98.47	17,714	98.08
Immunosuppressive drugs	43	0.67	86	0.70	6329	99.33	12,153	99.30
Sex								
Female	388	0.45	266	0.75	86,074	99.55	35,025	99.25
Male	220	0.42	168	0.65	52,212	99.58	25,543	99.35
Unknown	163	0.50	63	1.04	32,379	99.50	5969	98.96
Age group								
18 years below	13	0.51	4	0.27	2561	99.49	1465	99.73
18 to 64 years	239	0.42	149	0.60	56,722	99.58	24,888	99.40
65 years and above	322	0.75	267	1.00	42,621	99.25	26,381	99.00
Unknown	197	0.29	77	0.55	68,761	99.71	13,803	99.45
Reporter								
Healthcare professional	463	0.87	381	0.82	52,674	99.13	46,273	99.18
Non-healthcare professional	201	0.24	78	0.48	84,444	99.76	16,272	99.52
Unknown	107	0.32	38	0.94	33,547	99.68	3992	99.06
Report region								
North America	388	0.31	163	0.66	122,965	99.69	24,467	99.34
Europe	331	0.89	271	0.81	36,884	99.11	33,320	99.19
Asian	34	0.60	32	0.79	5657	99.40	4032	99.21
South America	3	0.22	2	0.24	1381	99.78	822	99.76
Africa	1	0.25	2	0.41	402	99.75	488	99.59
Oceania	0	0.00	2	0.18	986	100.00	1085	99.82
Unknown	14	0.58	25	1.06	2390	99.42	2323	98.94

FAERS, FDA adverse event reporting system; CDI, Clostridioides difficile infection; PPI, proton-pump inhibitor; PS, primary suspect drug; SS, secondary suspect drug.

**Table 2:** Disproportionality analysis of CDI events and primary suspect proton-pump inhibitors based on different analysis groups.

Analysis Groups and Drugs	CDI Evert Case		ROR Forest Plot	ROR		IC Forest Plot	IC	
	Number/n	Proportion/%		ROR	95%CI		IC	95%CI
No Case Excluded *	771	0.45	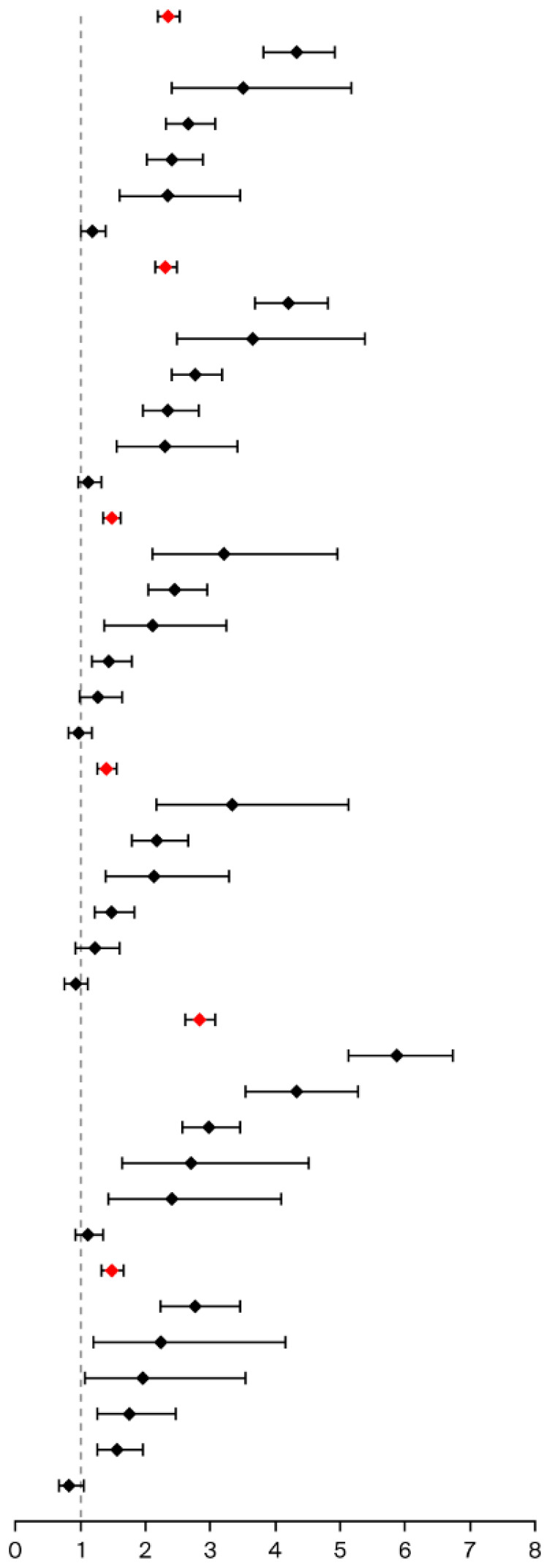	2.36	2.19 to 2.53	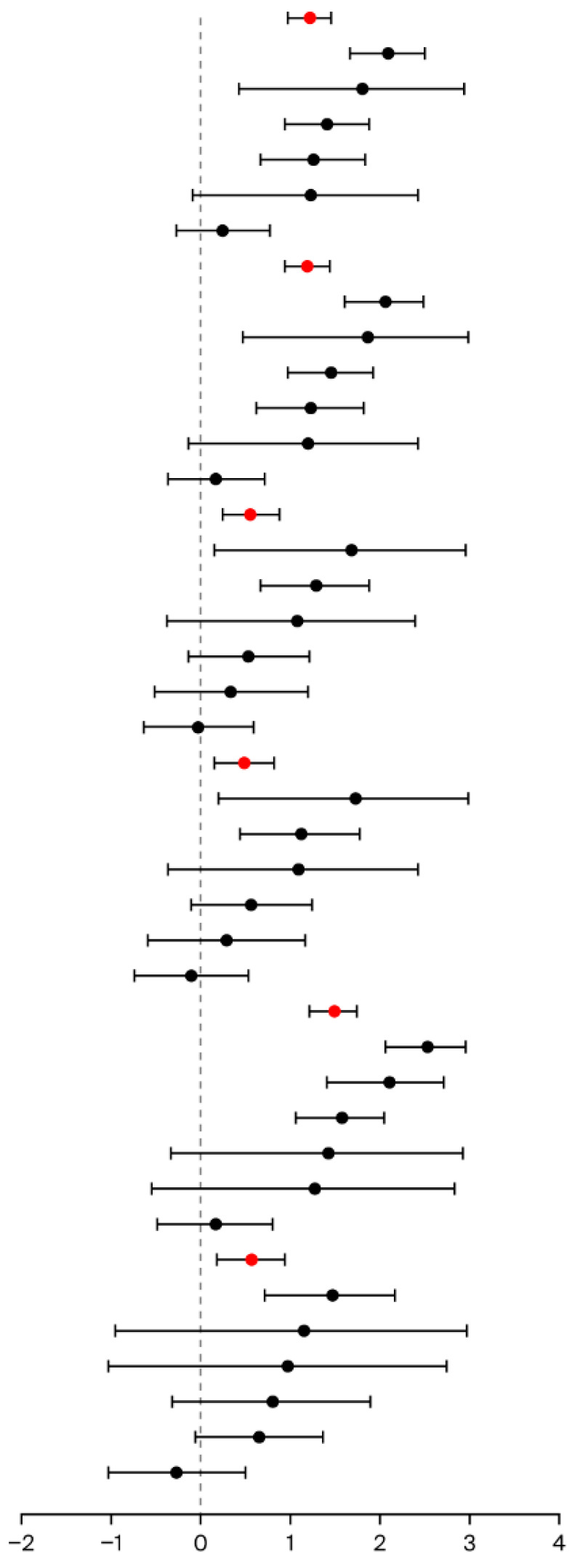	1.21	0.97 to 1.45
pantoprazole *	245	0.83		4.33	3.82 to 4.91		2.10	1.66 to 2.50
rabeprazole *	26	0.68		3.51	2.39 to 5.16		1.80	0.42 to 2.93
omeprazole *	193	0.51		2.67	2.32 to 3.08		1.41	0.93 to 1.87
lansoprazole *	124	0.47		2.41	2.02 to 2.88		126	0.66 to 1.83
dexlansoprazole	26	0.46		2.36	1.60 to 3.46		123	0.10 to 2.42
esomeprazole	156	0.23		1.18	1.01 to 1.38		0.24	−0.28 to 0.76
ISD Cases Excluded *	728	0.44		2.31	2.15 to 2.49		1.19	0.94 to 1.43
pantoprazole *	221	0.81		4.21	3.69 to 4.81		2.06	1.60 to 2.48
rabeprazole *	26	0.70		3.65	2.48 to 5.37		1.86	0.47 to 2.98
omeprazole *	192	0.53		2.77	2.40 to 3.19		1.46	0.97 to 1.92
lansoprazole *	118	0.45		2.35	1.96 to 2.82		1.23	0.61 to 1.81
dexlansoprazole	25	0.45		2.31	1.56 to 3.42		1.20	−0.15 to 2.41
esomeprazole	145	0.22		1.13	0.96 to 1.33		0.17	−0.37 to 0.71
ABD Cases Excluded *	422	0.28		1.47	1.34 to 1.62		0.55	023 to 0.87
rabeprazole *	21	0.62		3.22	2.10 to 4.95		1.68	0.15 to 2.94
pantoprazole *	117	0.47		2.45	2.04 to 2.94		128	0.66 to 1.87
dexlansoprazole	21	0.41		2.11	1.37 to 3.24		1.07	−0.39 to 2.39
omeprazole	93	0.28		1.45	1.18 to 1.78		0.53	−0.15 to 1.20
lansoprazole	57	0.25		1.27	0.98 to 1.64		0.34	−0.52 to 1.19
esomeprazole	112	0.19		0.98	0.81 to 1.18		−0.03	−0.64 to 0.59
ABD/ISD Cases Excluded *	390	0.27		1.40	1.27 to 1.55		0.48	0.15 to 0.81
rabeprazole *	21	0.64		3.34	2.17 to 5.13		1.73	0.19 to 2.98
pantoprazole *	97	0.42		2.18	1.78 to 2.66		1.12	0.44 to 1.76
dexlansoprazole	21	0.41		2.14	1.39 to 3.29		1.09	−0.37 to 2.41
omeprazole	92	0.29		1.48	1.21 to 1.82		0.56	−0.12 to 1.23
lansoprazole	54	0.24		1.22	0.93 to 1.60		0.29	−0.60 to 1.16
esomeprazole	104	0.18		0.93	0.76 to 1.12		−0.11	−0.75 to 0.53
RI Event Cases Excluded *	620	0.54		2.83	2.62 to 3.07		1.48	1.21 to 1.74
pantoprazole *	214	1.12		5.87	5.13 to 6.72		2.53	2.05 to 2.95
lansoprazole *	98	0.83		4.33	3.55 to 5.28		2.10	1.40 to 2.71
omeprazole *	176	0.57		2.99	2.57 to 3.46		1.57	1.06 to 2.04
rabeprazole	15	0.52		2.71	1.63 to 4.51		143	−0.34 to 2.92
dexlansoprazole	14	0.47		2.42	1.43 to 4.08		1.27	−0.55 to 2.82
esomeprazole	102	0.22		1.12	0.92 to 1.35		0.16	−0.49 to 0.80
ABD/ISD and RI Event Cases Excluded *	291	0.29		1.48	1.32 to 1.66		0.56	0.18 to 0.94
pantoprazole *	81	0.54		2.78	2.23 to 3.46		147	0.71 to 2.16
rabeprazole	10	0.43		2.24	1.20 to 4.16		1.16	−0.96 to 2.97
dexlansoprazole	11	0.38		1.96	1.08 to 3.54		0.97	−1.03 to 2.74
lansoprazole	34	0.34		1.75	1.25 to 2.46		0.81	−0.33 to 1.88
omeprazole	83	0.30		1.57	1.27 to 1.95		0.65	−0.07 to 1.35
esomeprazole	71	0.16		0.83	0.66 to 1.05		−0.27	−1.03 to 0.50

CDI, Clostridioides difficile infection; ROR, reporting odds ratio; 95%CI, 95% confidence interval; IC, information component; ISD, immunosuppressive drugs; ABD, antibacterial drugs; RI, renal injury; *, significant dementia disproportionality signal detected.

## Data Availability

The original data can be downloaded from the FDA adverse event reporting system public dashboard at: https://fis.fda.gov/extensions/FPD-QDE-FAERS/FPD-QDE-FAERS.html (accessed on 17 February 2024). The data presented in this study are available upon request from the corresponding author.

## Supplementary Materials

The following supporting information can be downloaded at: https://www.mdpi.com/article/10.3390/jcm15010230/s1, Figure S1. The reported trend of PPI cases in the FAERS database from 2004 to 2023. Figure S2. The reported trend of CDI cases associated with PPI treatment from the FAERS database. Table S1. Preferred terms for identifying Clostridioides difficile infection cases in FAERS database. Table S2. Identifying immunosuppressive drug cases using the Anatomical Therapeutic Chemical (ATC) classification system. Table S3. Identifying antibacterial drug cases using the Anatomical Therapeutic Chemical (ATC) classification system. Table S4. Preferred terms for identifying renal injury cases using HLGT search in FAERS database. Table S5. Two-by-two contingency table for reporting odds ratio analysis. Table S6. Disproportionality analysis of PPIs (primary or secondary suspect drug roles) and CDI. Table S7. Disproportionality analysis of PPIs (primary suspect drug role) and CDI based on different analyses and age groups. Table S8. Disproportionality analysis of PPIs (primary or secondary suspect drug roles) and CDI based on different analyses and age groups.

## Abbreviations

The following abbreviations are used in this manuscript:

PPI	Proton-pump inhibitor
CDI	Clostridioides difficile infection
FAERS	FDA adverse event reporting system
PS	Primary suspect drug
SS	Secondary suspect drug
DPA	Disproportionality analysis
ROR	Reporting odds ratio
IC	Information component
ISDs	Immunosuppressive drugs
ABDs	Antibacterial drugs
RI	Renal injury
DEMO	Patient demographic information table
DRUG	Drug information table
REAC	Adverse events information table
OUTC	Patient outcomes information table
RPSR	Report sources information table
THER	Drug therapy date information table
INDI	Drug indication table
