# Supplementary material for: A Vanished Association Between Proton Pump Inhibitors and Clostridioides Difficile Infection After Minimizing Bias
Source: J Clin Med. 2025 Dec 27;15(1):230. doi: 10.3390/jcm15010230 (PMC12787198; doi:10.3390/jcm15010230)

# A Vanished Association Between Proton Pump Inhibitors and Clostridioides Difficile Infection After Minimizing Bias

Bin Wu [1,2], Zhiyao He [1], Ting Xu [1, 2*]

## Supplementary Table S1

Preferred terms for identifying Clostridioides difficile infection cases in FAERS database.

| No. | PT code | Preferred term (PT) |
|---|---|---|
| 1 | 10054236 | Clostridium difficile infection |
| 2 | 10009657 | Clostridium difficile colitis |
| 3 | 10037128 | Pseudomembranous colitis |
| 4 | 10061043 | Clostridial infection |
| 5 | 10017898 | Gastroenteritis clostridial |
| 6 | 10058305 | Clostridium colitis |
| 7 | 10058852 | Clostridium bacteraemia |
| 8 | 10078496 | Clostridial sepsis |
| 9 | 10070027 | Clostridium test positive |

Supplementary Table S2

Identifying immunosuppressive drug cases using the Anatomical Therapeutic Chemical (ATC) classification system (code: L04 IMMUNOSUPPRESSANTS).

| Group | ATC code | Name |
|---|---|---|
| L04AA Selective immunosuppressants | L04AA03 | antilymphocyte immunoglobulin (horse) |
| L04AA Selective immunosuppressants | L04AA04 | antithymocyte immunoglobulin (rabbit) |
| L04AA Selective immunosuppressants | L04AA06 | mycophenolic acid |
| L04AA Selective immunosuppressants | L04AA15 | alefacept |
| L04AA Selective immunosuppressants | L04AA19 | gusperimus |
| L04AA Selective immunosuppressants | L04AA22 | abetimus |
| L04AA Selective immunosuppressants | L04AA24 | abatacept |
| L04AA Selective immunosuppressants | L04AA28 | belatacept |
| L04AA Selective immunosuppressants | L04AA32 | apremilast |
| L04AA Selective immunosuppressants | L04AA40 | cladribine |
| L04AA Selective immunosuppressants | L04AA41 | imlifidase |
| L04AA Selective immunosuppressants | L04AA48 | belumosudil |
| L04AA Selective immunosuppressants | L04AA58 | efgartigimod alfa |
| L04AA Selective immunosuppressants | L04AA60 | remibrutinib |
| L04AB Tumor necrosis factor alpha (TNF-α) inhibitors | L04AB01 | etanercept |
| L04AB Tumor necrosis factor alpha (TNF-α) inhibitors | L04AB02 | infliximab |
| L04AB Tumor necrosis factor alpha (TNF-α) inhibitors | L04AB03 | afelimomab |
| L04AB Tumor necrosis factor alpha (TNF-α) inhibitors | L04AB04 | adalimumab |
| L04AB Tumor necrosis factor alpha (TNF-α) inhibitors | L04AB05 | certolizumab pegol |
| L04AB Tumor necrosis factor alpha (TNF-α) inhibitors | L04AB06 | golimumab |
| L04AB Tumor necrosis factor alpha (TNF-α) inhibitors | L04AB07 | opinercept |
| L04AC Interleukin inhibitors | L04AC01 | daclizumab |
| L04AC Interleukin inhibitors | L04AC02 | basiliximab |
| L04AC Interleukin inhibitors | L04AC03 | anakinra |
| L04AC Interleukin inhibitors | L04AC04 | rilonacept |
| L04AC Interleukin inhibitors | L04AC05 | ustekinumab |
| L04AC Interleukin inhibitors | L04AC07 | tocilizumab |
| L04AC Interleukin inhibitors | L04AC08 | canakinumab |
| L04AC Interleukin inhibitors | L04AC09 | briakinumab |

| | | |
|---|---|---|
| L04AC Interleukin inhibitors | L04AC10 | secukinumab |
| L04AC Interleukin inhibitors | L04AC11 | siltuximab |
| L04AC Interleukin inhibitors | L04AC12 | brodalumab |
| L04AC Interleukin inhibitors | L04AC13 | ixekizumab |
| L04AC Interleukin inhibitors | L04AC14 | sarilumab |
| L04AC Interleukin inhibitors | L04AC15 | sirukumab |
| L04AC Interleukin inhibitors | L04AC16 | guselkumab |
| L04AC Interleukin inhibitors | L04AC17 | tildrakizumab |
| L04AC Interleukin inhibitors | L04AC18 | risankizumab |
| L04AC Interleukin inhibitors | L04AC19 | satralizumab |
| L04AC Interleukin inhibitors | L04AC20 | netakimab |
| L04AC Interleukin inhibitors | L04AC21 | bimekizumab |
| L04AC Interleukin inhibitors | L04AC22 | spesolimab |
| L04AC Interleukin inhibitors | L04AC23 | olokizumab |
| L04AC Interleukin inhibitors | L04AC24 | mirikizumab |
| L04AC Interleukin inhibitors | L04AC25 | levilimab |
| L04AC Interleukin inhibitors | L04AC26 | goflikicept |
| L04AD Calcineurin inhibitors | L04AD01 | ciclosporin |
| L04AD Calcineurin inhibitors | L04AD02 | tacrolimus |
| L04AD Calcineurin inhibitors | L04AD03 | voclosporin |
| L04AE Sphingosine-1-phosphate (S1P) receptor modulators | L04AE01 | fingolimod |
| L04AE Sphingosine-1-phosphate (S1P) receptor modulators | L04AE02 | ozanimod |
| L04AE Sphingosine-1-phosphate (S1P) receptor modulators | L04AE03 | siponimod |
| L04AE Sphingosine-1-phosphate (S1P) receptor modulators | L04AE04 | ponesimod |
| L04AE Sphingosine-1-phosphate (S1P) receptor modulators | L04AE05 | etrasimod |
| L04AF Janus-associated kinase (JAK) inhibitors | L04AF01 | tofacitinib |
| L04AF Janus-associated kinase (JAK) inhibitors | L04AF02 | baricitinib |
| L04AF Janus-associated kinase (JAK) inhibitors | L04AF03 | upadacitinib |
| L04AF Janus-associated kinase (JAK) inhibitors | L04AF04 | filgotinib |
| L04AF Janus-associated kinase (JAK) inhibitors | L04AF05 | itacitinib |
| L04AF Janus-associated kinase (JAK) inhibitors | L04AF06 | peficitinib |
| L04AF Janus-associated kinase (JAK) inhibitors | L04AF07 | deucravacitinib |

| | | |
|---|---|---|
| L04AF Janus-associated kinase (JAK) inhibitors | L04AF08 | ritlecitinib |
| L04AG Monoclonal antibodies | L04AG01 | muromonab-CD3 |
| L04AG Monoclonal antibodies | L04AG02 | efalizumab |
| L04AG Monoclonal antibodies | L04AG03 | natalizumab |
| L04AG Monoclonal antibodies | L04AG04 | belimumab |
| L04AG Monoclonal antibodies | L04AG05 | vedolizumab |
| L04AG Monoclonal antibodies | L04AG06 | alemtuzumab |
| L04AG Monoclonal antibodies | L04AG07 | begelomab |
| L04AG Monoclonal antibodies | L04AG08 | ocrelizumab |
| L04AG Monoclonal antibodies | L04AG09 | emapalumab |
| L04AG Monoclonal antibodies | L04AG10 | inebilizumab |
| L04AG Monoclonal antibodies | L04AG11 | anifrolumab |
| L04AG Monoclonal antibodies | L04AG12 | ofatumumab |
| L04AG Monoclonal antibodies | L04AG13 | teprotumumab |
| L04AG Monoclonal antibodies | L04AG14 | ublituximab |
| L04AG Monoclonal antibodies | L04AG15 | divozilimab |
| L04AG Monoclonal antibodies | L04AG16 | rozanolixizumab |
| L04AG Monoclonal antibodies | L04AG17 | seniprutug |
| L04AH Mammalian target of rapamycin (mTOR) kinase inhibitors | L04AH01 | sirolimus |
| L04AH Mammalian target of rapamycin (mTOR) kinase inhibitors | L04AH02 | everolimus |
| L04AJ Complement inhibitors | L04AJ01 | eculizumab |
| L04AJ Complement inhibitors | L04AJ02 | ravulizumab |
| L04AJ Complement inhibitors | L04AJ03 | pegcetacoplan |
| L04AJ Complement inhibitors | L04AJ04 | sutimlimab |
| L04AJ Complement inhibitors | L04AJ05 | avacopan |
| L04AJ Complement inhibitors | L04AJ06 | zilucoplan |
| L04AJ Complement inhibitors | L04AJ07 | crovalimab |
| L04AJ Complement inhibitors | L04AJ08 | iptacopan |
| L04AJ Complement inhibitors | L04AJ09 | danicopan |
| L04AJ Complement inhibitors | L04AJ10 | vilobelimab |
| L04AJ Complement inhibitors | L04AJ11 | pozelimab |
| L04AK Dihydroorotate dehydrogenase (DHODH) inhibitors | L04AK01 | leflunomide |
| L04AK Dihydroorotate dehydrogenase (DHODH) inhibitors | L04AK02 | teriflunomide |
| L04AX Other immunosuppressants | L04AX01 | azathioprine |
| L04AX Other immunosuppressants | L04AX02 | thalidomide |
| L04AX Other immunosuppressants | L04AX03 | methotrexate |
| L04AX Other immunosuppressants | L04AX04 | lenalidomide |
| L04AX Other immunosuppressants | L04AX05 | pirfenidone |
| L04AX Other immunosuppressants | L04AX06 | pomalidomide |

| | | |
|---|---|---|
| L04AX Other immunosuppressants | L04AX07 | dimethyl fumarate |
| L04AX Other immunosuppressants | L04AX08 | darvadstrocel |
| L04AX Other immunosuppressants | L04AX09 | diroximel fumarate |
| L04AX Other immunosuppressants | L04AX10 | tegomil fumarate |

**Supplementary Table S3**

Identifying antibacterial drug cases using the Anatomical Therapeutic Chemical (ATC) classification system (code: J01 ANTIBACTERIALS FOR SYSTEMIC USE).

| Group | ATC code | Name |
|---|---|---|
| J01A TETRACYCLINES | J01AA01 | demeclocycline |
| J01A TETRACYCLINES | J01AA02 | doxycycline |
| J01A TETRACYCLINES | J01AA03 | chlortetracycline |
| J01A TETRACYCLINES | J01AA04 | lymecycline |
| J01A TETRACYCLINES | J01AA05 | metacycline |
| J01A TETRACYCLINES | J01AA06 | oxytetracycline |
| J01A TETRACYCLINES | J01AA07 | tetracycline |
| J01A TETRACYCLINES | J01AA08 | minocycline |
| J01A TETRACYCLINES | J01AA09 | rolitetracycline |
| J01A TETRACYCLINES | J01AA10 | penimepicycline |
| J01A TETRACYCLINES | J01AA11 | clomocycline |
| J01A TETRACYCLINES | J01AA12 | tigecycline |
| J01A TETRACYCLINES | J01AA13 | eravacycline |
| J01A TETRACYCLINES | J01AA14 | sarecycline |
| J01A TETRACYCLINES | J01AA15 | omadacycline |
| J01A TETRACYCLINES | J01AA20 | combinations of tetracyclines |
| J01A TETRACYCLINES | J01AA56 | oxytetracycline, combinations |
| J01B AMPHENICOLS | J01BA01 | chloramphenicol |
| J01B AMPHENICOLS | J01BA02 | thiamphenicol |
| J01B AMPHENICOLS | J01BA52 | thiamphenicol, combinations |
| J01C BETA-LACTAM ANTIBACTERIALS, PENICILLINS | J01CA01 | ampicillin |
| J01C BETA-LACTAM ANTIBACTERIALS, PENICILLINS | J01CA02 | pivampicillin |
| J01C BETA-LACTAM ANTIBACTERIALS, PENICILLINS | J01CA03 | carbenicillin |
| J01C BETA-LACTAM ANTIBACTERIALS, PENICILLINS | J01CA04 | amoxicillin |
| J01C BETA-LACTAM ANTIBACTERIALS, PENICILLINS | J01CA05 | carindacillin |
| J01C BETA-LACTAM ANTIBACTERIALS, PENICILLINS | J01CA06 | bacampicillin |
| J01C BETA-LACTAM ANTIBACTERIALS, PENICILLINS | J01CA07 | epicillin |
| J01C BETA-LACTAM ANTIBACTERIALS, PENICILLINS | J01CA08 | pivmecillinam |

| | | |
|---|---|---|
| J01C BETA-LACTAM ANTIBACTERIALS, PENICILLINS | J01CA09 | azlocillin |
| J01C BETA-LACTAM ANTIBACTERIALS, PENICILLINS | J01CA10 | mezlocillin |
| J01C BETA-LACTAM ANTIBACTERIALS, PENICILLINS | J01CA11 | mecillinam |
| J01C BETA-LACTAM ANTIBACTERIALS, PENICILLINS | J01CA12 | piperacillin |
| J01C BETA-LACTAM ANTIBACTERIALS, PENICILLINS | J01CA13 | ticarcillin |
| J01C BETA-LACTAM ANTIBACTERIALS, PENICILLINS | J01CA14 | metampicillin |
| J01C BETA-LACTAM ANTIBACTERIALS, PENICILLINS | J01CA15 | talampicillin |
| J01C BETA-LACTAM ANTIBACTERIALS, PENICILLINS | J01CA16 | sulbenicillin |
| J01C BETA-LACTAM ANTIBACTERIALS, PENICILLINS | J01CA17 | temocillin |
| J01C BETA-LACTAM ANTIBACTERIALS, PENICILLINS | J01CA18 | hetacillin |
| J01C BETA-LACTAM ANTIBACTERIALS, PENICILLINS | J01CA19 | aspoxicillin |
| J01C BETA-LACTAM ANTIBACTERIALS, PENICILLINS | J01CA20 | combinations |
| J01C BETA-LACTAM ANTIBACTERIALS, PENICILLINS | J01CA51 | ampicillin, combinations |
| J01C BETA-LACTAM ANTIBACTERIALS, PENICILLINS | J01CE01 | benzylpenicillin |
| J01C BETA-LACTAM ANTIBACTERIALS, PENICILLINS | J01CE02 | phenoxymethylpenicillin |
| J01C BETA-LACTAM ANTIBACTERIALS, PENICILLINS | J01CE03 | propicillin |
| J01C BETA-LACTAM ANTIBACTERIALS, PENICILLINS | J01CE04 | azidocillin |
| J01C BETA-LACTAM ANTIBACTERIALS, PENICILLINS | J01CE05 | pheneticillin |
| J01C BETA-LACTAM ANTIBACTERIALS, PENICILLINS | J01CE06 | penamecillin |
| J01C BETA-LACTAM ANTIBACTERIALS, PENICILLINS | J01CE07 | clometocillin |
| J01C BETA-LACTAM ANTIBACTERIALS, PENICILLINS | J01CE08 | benzathine benzylpenicillin |
| J01C BETA-LACTAM ANTIBACTERIALS, PENICILLINS | J01CE09 | procaine benzylpenicillin |

| | | |
|---|---|---|
| J01C BETA-LACTAM ANTIBACTERIALS, PENICILLINS | J01CE10 | benzathine phenoxymethylpenicillin |
| J01C BETA-LACTAM ANTIBACTERIALS, PENICILLINS | J01CE30 | combinations |
| J01C BETA-LACTAM ANTIBACTERIALS, PENICILLINS | J01CF01 | dicloxacillin |
| J01C BETA-LACTAM ANTIBACTERIALS, PENICILLINS | J01CF02 | cloxacillin |
| J01C BETA-LACTAM ANTIBACTERIALS, PENICILLINS | J01CF03 | meticillin |
| J01C BETA-LACTAM ANTIBACTERIALS, PENICILLINS | J01CF04 | oxacillin |
| J01C BETA-LACTAM ANTIBACTERIALS, PENICILLINS | J01CF05 | flucloxacillin |
| J01C BETA-LACTAM ANTIBACTERIALS, PENICILLINS | J01CF06 | nafcillin |
| J01C BETA-LACTAM ANTIBACTERIALS, PENICILLINS | J01CG01 | sulbactam |
| J01C BETA-LACTAM ANTIBACTERIALS, PENICILLINS | J01CG02 | tazobactam |
| J01C BETA-LACTAM ANTIBACTERIALS, PENICILLINS | J01CR01 | ampicillin and beta-lactamase inhibitor |
| J01C BETA-LACTAM ANTIBACTERIALS, PENICILLINS | J01CR02 | amoxicillin and beta-lactamase inhibitor |
| J01C BETA-LACTAM ANTIBACTERIALS, PENICILLINS | J01CR03 | ticarcillin and beta-lactamase inhibitor |
| J01C BETA-LACTAM ANTIBACTERIALS, PENICILLINS | J01CR04 | sultamicillin |
| J01C BETA-LACTAM ANTIBACTERIALS, PENICILLINS | J01CR05 | piperacillin and beta-lactamase inhibitor |
| J01C BETA-LACTAM ANTIBACTERIALS, PENICILLINS | J01CR50 | combinations of penicillins |
| J01D OTHER BETA-LACTAM ANTIBACTERIALS | J01DB01 | cefalexin |
| J01D OTHER BETA-LACTAM ANTIBACTERIALS | J01DB02 | cefaloridine |
| J01D OTHER BETA-LACTAM ANTIBACTERIALS | J01DB03 | cefalotin |
| J01D OTHER BETA-LACTAM ANTIBACTERIALS | J01DB04 | cefazolin |
| J01D OTHER BETA-LACTAM ANTIBACTERIALS | J01DB05 | cefadroxil |
| J01D OTHER BETA-LACTAM ANTIBACTERIALS | J01DB06 | cefazedone |

| | | |
|---|---|---|
| J01D OTHER BETA-LACTAM ANTIBACTERIALS | J01DB07 | cefatrizine |
| J01D OTHER BETA-LACTAM ANTIBACTERIALS | J01DB08 | cefapirin |
| J01D OTHER BETA-LACTAM ANTIBACTERIALS | J01DB09 | cefradine |
| J01D OTHER BETA-LACTAM ANTIBACTERIALS | J01DB10 | cefacetrile |
| J01D OTHER BETA-LACTAM ANTIBACTERIALS | J01DB11 | cefroxadine |
| J01D OTHER BETA-LACTAM ANTIBACTERIALS | J01DB12 | ceftezole |
| J01D OTHER BETA-LACTAM ANTIBACTERIALS | J01DC01 | cefoxitin |
| J01D OTHER BETA-LACTAM ANTIBACTERIALS | J01DC02 | cefuroxime |
| J01D OTHER BETA-LACTAM ANTIBACTERIALS | J01DC03 | cefamandole |
| J01D OTHER BETA-LACTAM ANTIBACTERIALS | J01DC04 | cefaclor |
| J01D OTHER BETA-LACTAM ANTIBACTERIALS | J01DC05 | cefotetan |
| J01D OTHER BETA-LACTAM ANTIBACTERIALS | J01DC06 | cefonicid |
| J01D OTHER BETA-LACTAM ANTIBACTERIALS | J01DC07 | cefotiam |
| J01D OTHER BETA-LACTAM ANTIBACTERIALS | J01DC08 | loracarbef |
| J01D OTHER BETA-LACTAM ANTIBACTERIALS | J01DC09 | cefmetazole |
| J01D OTHER BETA-LACTAM ANTIBACTERIALS | J01DC10 | cefprozil |
| J01D OTHER BETA-LACTAM ANTIBACTERIALS | J01DC11 | ceforanide |
| J01D OTHER BETA-LACTAM ANTIBACTERIALS | J01DC12 | cefminox |
| J01D OTHER BETA-LACTAM ANTIBACTERIALS | J01DC13 | cefbuperazone |
| J01D OTHER BETA-LACTAM ANTIBACTERIALS | J01DC14 | flomoxef |
| J01D OTHER BETA-LACTAM ANTIBACTERIALS | J01DC52 | cefuroxime and beta-lactamase inhibitor |
| J01D OTHER BETA-LACTAM ANTIBACTERIALS | J01DD01 | cefotaxime |

| | | |
|---|---|---|
| J01D OTHER BETA-LACTAM ANTIBACTERIALS | J01DD02 | ceftazidime |
| J01D OTHER BETA-LACTAM ANTIBACTERIALS | J01DD03 | cefsulodin |
| J01D OTHER BETA-LACTAM ANTIBACTERIALS | J01DD04 | ceftriaxone |
| J01D OTHER BETA-LACTAM ANTIBACTERIALS | J01DD05 | cefmenoxime |
| J01D OTHER BETA-LACTAM ANTIBACTERIALS | J01DD06 | latamoxef |
| J01D OTHER BETA-LACTAM ANTIBACTERIALS | J01DD07 | ceftizoxime |
| J01D OTHER BETA-LACTAM ANTIBACTERIALS | J01DD08 | cefixime |
| J01D OTHER BETA-LACTAM ANTIBACTERIALS | J01DD09 | cefodizime |
| J01D OTHER BETA-LACTAM ANTIBACTERIALS | J01DD10 | cefetamet |
| J01D OTHER BETA-LACTAM ANTIBACTERIALS | J01DD11 | cefpiramide |
| J01D OTHER BETA-LACTAM ANTIBACTERIALS | J01DD12 | cefoperazone |
| J01D OTHER BETA-LACTAM ANTIBACTERIALS | J01DD13 | cefpodoxime |
| J01D OTHER BETA-LACTAM ANTIBACTERIALS | J01DD14 | ceftibuten |
| J01D OTHER BETA-LACTAM ANTIBACTERIALS | J01DD15 | cefdinir |
| J01D OTHER BETA-LACTAM ANTIBACTERIALS | J01DD16 | cefditoren |
| J01D OTHER BETA-LACTAM ANTIBACTERIALS | J01DD17 | cefcapene |
| J01D OTHER BETA-LACTAM ANTIBACTERIALS | J01DD18 | cefteram |
| J01D OTHER BETA-LACTAM ANTIBACTERIALS | J01DD51 | cefotaxime and beta-lactamase inhibitor |
| J01D OTHER BETA-LACTAM ANTIBACTERIALS | J01DD52 | ceftazidime and beta-lactamase inhibitor |
| J01D OTHER BETA-LACTAM ANTIBACTERIALS | J01DD54 | ceftriaxone, combinations |
| J01D OTHER BETA-LACTAM ANTIBACTERIALS | J01DD58 | cefixime and beta-lactamase inhibitor |
| J01D OTHER BETA-LACTAM ANTIBACTERIALS | J01DD62 | cefoperazone and beta-lactamase inhibitor |

| | | |
|---|---|---|
| J01D OTHER BETA-LACTAM ANTIBACTERIALS | J01DD63 | ceftriaxone and beta-lactamase inhibitor |
| J01D OTHER BETA-LACTAM ANTIBACTERIALS | J01DD64 | cefpodoxime and beta-lactamase inhibitor |
| J01D OTHER BETA-LACTAM ANTIBACTERIALS | J01DE01 | cefepime |
| J01D OTHER BETA-LACTAM ANTIBACTERIALS | J01DE02 | cefpirome |
| J01D OTHER BETA-LACTAM ANTIBACTERIALS | J01DE03 | cefozopran |
| J01D OTHER BETA-LACTAM ANTIBACTERIALS | J01DE51 | cefepime and beta-lactamase inhibitor |
| J01D OTHER BETA-LACTAM ANTIBACTERIALS | J01DF01 | aztreonam |
| J01D OTHER BETA-LACTAM ANTIBACTERIALS | J01DF02 | carumonam |
| J01D OTHER BETA-LACTAM ANTIBACTERIALS | J01DF51 | aztreonam and beta-lactamase inhibitor |
| J01D OTHER BETA-LACTAM ANTIBACTERIALS | J01DH02 | meropenem |
| J01D OTHER BETA-LACTAM ANTIBACTERIALS | J01DH03 | ertapenem |
| J01D OTHER BETA-LACTAM ANTIBACTERIALS | J01DH04 | doripenem |
| J01D OTHER BETA-LACTAM ANTIBACTERIALS | J01DH05 | biapenem |
| J01D OTHER BETA-LACTAM ANTIBACTERIALS | J01DH06 | tebipenem pivoxil |
| J01D OTHER BETA-LACTAM ANTIBACTERIALS | J01DH51 | imipenem and cilastatin |
| J01D OTHER BETA-LACTAM ANTIBACTERIALS | J01DH52 | meropenem and vaborbactam |
| J01D OTHER BETA-LACTAM ANTIBACTERIALS | J01DH55 | panipenem and betamipron |
| J01D OTHER BETA-LACTAM ANTIBACTERIALS | J01DH56 | imipenem, cilastatin and relebactam |
| J01D OTHER BETA-LACTAM ANTIBACTERIALS | J01DI01 | ceftobiprole medocaril |
| J01D OTHER BETA-LACTAM ANTIBACTERIALS | J01DI02 | ceftaroline fosamil |
| J01D OTHER BETA-LACTAM ANTIBACTERIALS | J01DI03 | faropenem |
| J01D OTHER BETA-LACTAM ANTIBACTERIALS | J01DI04 | cefiderocol |

| | | |
|---|---|---|
| J01D OTHER BETA-LACTAM ANTIBACTERIALS | J01DI54 | ceftolozane and beta-lactamase inhibitor |
| J01E SULFONAMIDES AND TRIMETHOPRIM | J01EA01 | trimethoprim |
| J01E SULFONAMIDES AND TRIMETHOPRIM | J01EA02 | brodimoprim |
| J01E SULFONAMIDES AND TRIMETHOPRIM | J01EA03 | iclaprim |
| J01E SULFONAMIDES AND TRIMETHOPRIM | J01EB01 | sulfaisodimidine |
| J01E SULFONAMIDES AND TRIMETHOPRIM | J01EB02 | sulfamethizole |
| J01E SULFONAMIDES AND TRIMETHOPRIM | J01EB03 | sulfadimidine |
| J01E SULFONAMIDES AND TRIMETHOPRIM | J01EB04 | sulfapyridine |
| J01E SULFONAMIDES AND TRIMETHOPRIM | J01EB05 | sulfafurazole |
| J01E SULFONAMIDES AND TRIMETHOPRIM | J01EB06 | sulfanilamide |
| J01E SULFONAMIDES AND TRIMETHOPRIM | J01EB07 | sulfathiazole |
| J01E SULFONAMIDES AND TRIMETHOPRIM | J01EB08 | sulfathiourea |
| J01E SULFONAMIDES AND TRIMETHOPRIM | J01EB20 | combinations |
| J01E SULFONAMIDES AND TRIMETHOPRIM | J01EC01 | sulfamethoxazole |
| J01E SULFONAMIDES AND TRIMETHOPRIM | J01EC02 | sulfadiazine |
| J01E SULFONAMIDES AND TRIMETHOPRIM | J01EC03 | sulfamoxole |
| J01E SULFONAMIDES AND TRIMETHOPRIM | J01EC20 | combinations |
| J01E SULFONAMIDES AND TRIMETHOPRIM | J01ED01 | sulfadimethoxine |
| J01E SULFONAMIDES AND TRIMETHOPRIM | J01ED02 | sulfalene |
| J01E SULFONAMIDES AND TRIMETHOPRIM | J01ED03 | sulfametomidine |
| J01E SULFONAMIDES AND TRIMETHOPRIM | J01ED04 | sulfametoxydiazine |
| J01E SULFONAMIDES AND TRIMETHOPRIM | J01ED05 | sulfamethoxypyridazine |

| | | |
|---|---|---|
| J01E SULFONAMIDES AND TRIMETHOPRIM | J01ED06 | sulfaperin |
| J01E SULFONAMIDES AND TRIMETHOPRIM | J01ED07 | sulfamerazine |
| J01E SULFONAMIDES AND TRIMETHOPRIM | J01ED08 | sulfaphenazole |
| J01E SULFONAMIDES AND TRIMETHOPRIM | J01ED09 | sulfamazone |
| J01E SULFONAMIDES AND TRIMETHOPRIM | J01ED20 | combinations |
| J01E SULFONAMIDES AND TRIMETHOPRIM | J01EE01 | sulfamethoxazole and trimethoprim |
| J01E SULFONAMIDES AND TRIMETHOPRIM | J01EE02 | sulfadiazine and trimethoprim |
| J01E SULFONAMIDES AND TRIMETHOPRIM | J01EE03 | sulfametrole and trimethoprim |
| J01E SULFONAMIDES AND TRIMETHOPRIM | J01EE04 | sulfamoxole and trimethoprim |
| J01E SULFONAMIDES AND TRIMETHOPRIM | J01EE05 | sulfadimidine and trimethoprim |
| J01E SULFONAMIDES AND TRIMETHOPRIM | J01EE06 | sulfadiazine and tetroxoprim |
| J01E SULFONAMIDES AND TRIMETHOPRIM | J01EE07 | sulfamerazine and trimethoprim |
| J01F MACROLIDES, LINCOSAMIDES AND STREPTOGRAMINS | J01FA01 | erythromycin |
| J01F MACROLIDES, LINCOSAMIDES AND STREPTOGRAMINS | J01FA02 | spiramycin |
| J01F MACROLIDES, LINCOSAMIDES AND STREPTOGRAMINS | J01FA03 | midecamycin |
| J01F MACROLIDES, LINCOSAMIDES AND STREPTOGRAMINS | J01FA05 | oleandomycin |
| J01F MACROLIDES, LINCOSAMIDES AND STREPTOGRAMINS | J01FA06 | roxithromycin |
| J01F MACROLIDES, LINCOSAMIDES AND STREPTOGRAMINS | J01FA07 | josamycin |

| | | |
|---|---|---|
| J01F MACROLIDES, LINCOSAMIDES AND STREPTOGRAMINS | J01FA08 | troleandomycin |
| J01F MACROLIDES, LINCOSAMIDES AND STREPTOGRAMINS | J01FA09 | clarithromycin |
| J01F MACROLIDES, LINCOSAMIDES AND STREPTOGRAMINS | J01FA10 | azithromycin |
| J01F MACROLIDES, LINCOSAMIDES AND STREPTOGRAMINS | J01FA11 | miocamycin |
| J01F MACROLIDES, LINCOSAMIDES AND STREPTOGRAMINS | J01FA12 | rokitamycin |
| J01F MACROLIDES, LINCOSAMIDES AND STREPTOGRAMINS | J01FA13 | dirithromycin |
| J01F MACROLIDES, LINCOSAMIDES AND STREPTOGRAMINS | J01FA14 | flurithromycin |
| J01F MACROLIDES, LINCOSAMIDES AND STREPTOGRAMINS | J01FA15 | telithromycin |
| J01F MACROLIDES, LINCOSAMIDES AND STREPTOGRAMINS | J01FA16 | solithromycin |
| J01F MACROLIDES, LINCOSAMIDES AND STREPTOGRAMINS | J01FF01 | clindamycin |
| J01F MACROLIDES, LINCOSAMIDES AND STREPTOGRAMINS | J01FF02 | lincomycin |
| J01F MACROLIDES, LINCOSAMIDES AND STREPTOGRAMINS | J01FG01 | pristinamycin |
| J01F MACROLIDES, LINCOSAMIDES AND STREPTOGRAMINS | J01FG02 | quinupristin/dalfopristin |
| J01G AMINOGLYCOSIDE ANTIBACTERIALS | J01GA01 | streptomycin |
| J01G AMINOGLYCOSIDE ANTIBACTERIALS | J01GA02 | streptoduocin |

| | | |
|---|---|---|
| J01G AMINOGLYCOSIDE ANTIBACTERIALS | J01GB01 | tobramycin |
| J01G AMINOGLYCOSIDE ANTIBACTERIALS | J01GB03 | gentamicin |
| J01G AMINOGLYCOSIDE ANTIBACTERIALS | J01GB04 | kanamycin |
| J01G AMINOGLYCOSIDE ANTIBACTERIALS | J01GB05 | neomycin |
| J01G AMINOGLYCOSIDE ANTIBACTERIALS | J01GB06 | amikacin |
| J01G AMINOGLYCOSIDE ANTIBACTERIALS | J01GB07 | netilmicin |
| J01G AMINOGLYCOSIDE ANTIBACTERIALS | J01GB08 | sisomicin |
| J01G AMINOGLYCOSIDE ANTIBACTERIALS | J01GB09 | dibekacin |
| J01G AMINOGLYCOSIDE ANTIBACTERIALS | J01GB10 | ribostamycin |
| J01G AMINOGLYCOSIDE ANTIBACTERIALS | J01GB11 | isepamicin |
| J01G AMINOGLYCOSIDE ANTIBACTERIALS | J01GB12 | arbekacin |
| J01G AMINOGLYCOSIDE ANTIBACTERIALS | J01GB13 | bekanamycin |
| J01G AMINOGLYCOSIDE ANTIBACTERIALS | J01GB14 | plazomicin |
| J01M QUINOLONE ANTIBACTERIALS | J01MA01 | ofloxacin |
| J01M QUINOLONE ANTIBACTERIALS | J01MA02 | ciprofloxacin |
| J01M QUINOLONE ANTIBACTERIALS | J01MA03 | pefloxacin |
| J01M QUINOLONE ANTIBACTERIALS | J01MA04 | enoxacin |
| J01M QUINOLONE ANTIBACTERIALS | J01MA05 | temafloxacin |
| J01M QUINOLONE ANTIBACTERIALS | J01MA06 | norfloxacin |
| J01M QUINOLONE ANTIBACTERIALS | J01MA07 | lomefloxacin |
| J01M QUINOLONE ANTIBACTERIALS | J01MA08 | fleroxacin |
| J01M QUINOLONE ANTIBACTERIALS | J01MA09 | sparfloxacin |

| | | |
|---|---|---|
| J01M QUINOLONE ANTIBACTERIALS | J01MA10 | rufloxacin |
| J01M QUINOLONE ANTIBACTERIALS | J01MA11 | grepafloxacin |
| J01M QUINOLONE ANTIBACTERIALS | J01MA12 | levofloxacin |
| J01M QUINOLONE ANTIBACTERIALS | J01MA13 | trovafloxacin |
| J01M QUINOLONE ANTIBACTERIALS | J01MA14 | moxifloxacin |
| J01M QUINOLONE ANTIBACTERIALS | J01MA15 | gemifloxacin |
| J01M QUINOLONE ANTIBACTERIALS | J01MA16 | gatifloxacin |
| J01M QUINOLONE ANTIBACTERIALS | J01MA17 | prulifloxacin |
| J01M QUINOLONE ANTIBACTERIALS | J01MA18 | pazufloxacin |
| J01M QUINOLONE ANTIBACTERIALS | J01MA19 | garenoxacin |
| J01M QUINOLONE ANTIBACTERIALS | J01MA21 | sitafloxacin |
| J01M QUINOLONE ANTIBACTERIALS | J01MA22 | tosufloxacin |
| J01M QUINOLONE ANTIBACTERIALS | J01MA23 | delafloxacin |
| J01M QUINOLONE ANTIBACTERIALS | J01MA24 | levonadifloxacin |
| J01M QUINOLONE ANTIBACTERIALS | J01MA25 | lascufloxacin |
| J01M QUINOLONE ANTIBACTERIALS | J01MB01 | rosoxacin |
| J01M QUINOLONE ANTIBACTERIALS | J01MB02 | nalidixic acid |
| J01M QUINOLONE ANTIBACTERIALS | J01MB03 | piromidic acid |
| J01M QUINOLONE ANTIBACTERIALS | J01MB04 | pipemidic acid |
| J01M QUINOLONE ANTIBACTERIALS | J01MB05 | oxolinic acid |
| J01M QUINOLONE ANTIBACTERIALS | J01MB06 | cinoxacin |
| J01M QUINOLONE ANTIBACTERIALS | J01MB07 | flumequine |

| | | |
|---|---|---|
| J01M QUINOLONE ANTIBACTERIALS | J01MB08 | nemonoxacin |
| J01R COMBINATIONS OF ANTIBACTERIALS | J01RA01 | penicillins, combinations with other antibacterials |
| J01R COMBINATIONS OF ANTIBACTERIALS | J01RA02 | sulfonamides, combinations with other antibacterials (excl. trimethoprim) |
| J01R COMBINATIONS OF ANTIBACTERIALS | J01RA03 | cefuroxime and metronidazole |
| J01R COMBINATIONS OF ANTIBACTERIALS | J01RA04 | spiramycin and metronidazole |
| J01R COMBINATIONS OF ANTIBACTERIALS | J01RA05 | levofloxacin and ornidazole |
| J01R COMBINATIONS OF ANTIBACTERIALS | J01RA06 | cefepime and amikacin |
| J01R COMBINATIONS OF ANTIBACTERIALS | J01RA07 | azithromycin, fluconazole and secnidazole |
| J01R COMBINATIONS OF ANTIBACTERIALS | J01RA08 | tetracycline and oleandomycin |
| J01R COMBINATIONS OF ANTIBACTERIALS | J01RA09 | ofloxacin and ornidazole |
| J01R COMBINATIONS OF ANTIBACTERIALS | J01RA10 | ciprofloxacin and metronidazole |
| J01R COMBINATIONS OF ANTIBACTERIALS | J01RA11 | ciprofloxacin and tinidazole |
| J01R COMBINATIONS OF ANTIBACTERIALS | J01RA12 | ciprofloxacin and ornidazole |
| J01R COMBINATIONS OF ANTIBACTERIALS | J01RA13 | norfloxacin and tinidazole |
| J01R COMBINATIONS OF ANTIBACTERIALS | J01RA14 | norfloxacin and metronidazole |
| J01R COMBINATIONS OF ANTIBACTERIALS | J01RA15 | cefixime and ornidazole |
| J01R COMBINATIONS OF ANTIBACTERIALS | J01RA16 | cefixime and azithromycin |
| J01R COMBINATIONS OF ANTIBACTERIALS | J01RA17 | ofloxacin and nitazoxanide |
| J01R COMBINATIONS OF ANTIBACTERIALS | J01RA18 | ofloxacin and tinidazole |
| J01R COMBINATIONS OF ANTIBACTERIALS | J01RA19 | tetracycline and nystatin |
| J01X OTHER ANTIBACTERIALS | J01XA01 | vancomycin |
| J01X OTHER ANTIBACTERIALS | J01XA02 | teicoplanin |
| J01X OTHER ANTIBACTERIALS | J01XA03 | telavancin |
| J01X OTHER ANTIBACTERIALS | J01XA04 | dalbavancin |

| | | |
|---|---|---|
| J01X OTHER ANTIBACTERIALS | J01XA05 | oritavancin |
| J01X OTHER ANTIBACTERIALS | J01XB01 | colistin |
| J01X OTHER ANTIBACTERIALS | J01XB02 | polymyxin B |
| J01X OTHER ANTIBACTERIALS | J01XC01 | fusidic acid |
| J01X OTHER ANTIBACTERIALS | J01XD01 | metronidazole |
| J01X OTHER ANTIBACTERIALS | J01XD02 | tinidazole |
| J01X OTHER ANTIBACTERIALS | J01XD03 | ornidazole |
| J01X OTHER ANTIBACTERIALS | J01XE01 | nitrofurantoin |
| J01X OTHER ANTIBACTERIALS | J01XE02 | nifurtoinol |
| J01X OTHER ANTIBACTERIALS | J01XE03 | furazidin |
| J01X OTHER ANTIBACTERIALS | J01XE51 | nitrofurantoin, combinations |
| J01X OTHER ANTIBACTERIALS | J01XX01 | fosfomycin |
| J01X OTHER ANTIBACTERIALS | J01XX02 | xibornol |
| J01X OTHER ANTIBACTERIALS | J01XX03 | clofoctol |
| J01X OTHER ANTIBACTERIALS | J01XX04 | spectinomycin |
| J01X OTHER ANTIBACTERIALS | J01XX05 | methenamine |
| J01X OTHER ANTIBACTERIALS | J01XX06 | mandelic acid |
| J01X OTHER ANTIBACTERIALS | J01XX07 | nitroxoline |
| J01X OTHER ANTIBACTERIALS | J01XX08 | linezolid |
| J01X OTHER ANTIBACTERIALS | J01XX09 | daptomycin |
| J01X OTHER ANTIBACTERIALS | J01XX10 | bacitracin |
| J01X OTHER ANTIBACTERIALS | J01XX11 | tedizolid |
| J01X OTHER ANTIBACTERIALS | J01XX12 | lefamulin |
| J01X OTHER ANTIBACTERIALS | J01XX13 | gepotidacin |

## Supplementary Table S4

Preferred terms for identifying renal injury cases using HLGT (code: 10029149, 1003843) search in FAERS database.

| No. | PT code | Preferred term (PT) |
|---|---|---|
| 1 | 10002847 | Anuria |
| 2 | 10003885 | Azotaemia |
| 3 | 10012660 | Diabetic end stage renal disease |
| 4 | 10018362 | Glomerular vascular disorder |
| 5 | 10018364 | Glomerulonephritis |
| 6 | 10018366 | Glomerulonephritis acute |
| 7 | 10018367 | Glomerulonephritis chronic |
| 8 | 10018370 | Glomerulonephritis membranoproliferative |
| 9 | 10018372 | Glomerulonephritis membranous |
| 10 | 10018374 | Glomerulonephritis minimal lesion |
| 11 | 10018376 | Glomerulonephritis proliferative |
| 12 | 10018378 | Glomerulonephritis rapidly progressive |
| 13 | 10018620 | Goodpasture's syndrome |
| 14 | 10020524 | Hydronephrosis |
| 15 | 10020586 | Hypercalcaemic nephropathy |
| 16 | 10021263 | IgA nephropathy |
| 17 | 10022530 | Intercapillary glomerulosclerosis |
| 18 | 10023421 | Kidney fibrosis |
| 19 | 10023423 | Kidney hypermobility |
| 20 | 10023435 | Kidney small |
| 21 | 10025140 | Lupus nephritis |
| 22 | 10026674 | Malignant renal hypertension |
| 23 | 10029117 | Nephritis |
| 24 | 10029120 | Nephritis allergic |
| 25 | 10029132 | Nephritis haemorrhagic |
| 26 | 10029147 | Nephrogenic diabetes insipidus |
| 27 | 10029151 | Nephropathy |
| 28 | 10029155 | Nephropathy toxic |
| 29 | 10029158 | Nephroptosis |
| 30 | 10029159 | Nephrosclerosis |
| 31 | 10029164 | Nephrotic syndrome |
| 32 | 10030302 | Oliguria |
| 33 | 10034232 | Pelvi-ureteric obstruction |
| 34 | 10036303 | Post streptococcal glomerulonephritis |
| 35 | 10038357 | Renal amyloidosis |
| 36 | 10038366 | Renal aneurysm |
| 37 | 10038372 | Renal arteriosclerosis |

| | | |
|---|---|---|
| 38 | 10038373 | Renal arteritis |
| 39 | 10038377 | Renal artery hyperplasia |
| 40 | 10038378 | Renal artery stenosis |
| 41 | 10038380 | Renal artery thrombosis |
| 42 | 10038381 | Renal atrophy |
| 43 | 10038422 | Renal cortical necrosis |
| 44 | 10038423 | Renal cyst |
| 45 | 10038428 | Renal disorder |
| 46 | 10038435 | Renal failure |
| 47 | 10038447 | Renal failure neonatal |
| 48 | 10038457 | Renal glycosuria |
| 49 | 10038459 | Renal haematoma |
| 50 | 10038460 | Renal haemorrhage |
| 51 | 10038464 | Renal hypertension |
| 52 | 10038468 | Renal hypertrophy |
| 53 | 10038470 | Renal infarct |
| 54 | 10038481 | Renal necrosis |
| 55 | 10038491 | Renal papillary necrosis |
| 56 | 10038535 | Renal tubular acidosis |
| 57 | 10038536 | Renal tubular atrophy |
| 58 | 10038537 | Renal tubular disorder |
| 59 | 10038540 | Renal tubular necrosis |
| 60 | 10038546 | Renal vasculitis |
| 61 | 10038547 | Renal vein embolism |
| 62 | 10038548 | Renal vein thrombosis |
| 63 | 10038553 | Renal vessel disorder |
| 64 | 10046337 | Urate nephropathy |
| 65 | 10048302 | Tubulointerstitial nephritis |
| 66 | 10048469 | Kidney enlargement |
| 67 | 10048988 | Renal artery occlusion |
| 68 | 10049739 | Renal artery fibromuscular dysplasia |
| 69 | 10049776 | Renal impairment neonatal |
| 70 | 10049778 | Neonatal anuria |
| 71 | 10049814 | Renal cyst ruptured |
| 72 | 10049942 | Renal artery dissection |
| 73 | 10050335 | Renal tubular dysfunction |
| 74 | 10050702 | Crush syndrome |
| 75 | 10051467 | Nephrectasia |
| 76 | 10051920 | Glomerulonephropathy |
| 77 | 10051985 | Renal pelvis fistula |
| 78 | 10052242 | Nephroangiosclerosis |
| 79 | 10052607 | Fanconi syndrome acquired |
| 80 | 10054832 | Diffuse mesangial sclerosis |
| 81 | 10055171 | Hypertensive nephropathy |

| | | |
|---|---|---|
| 82 | 10056277 | Pancreatorenal syndrome |
| 83 | 10056293 | Renal vein occlusion |
| 84 | 10056505 | Renal disorder in pregnancy |
| 85 | 10056609 | Uraemia odour |
| 86 | 10057345 | Renal lipomatosis |
| 87 | 10057399 | Calyceal diverticulum |
| 88 | 10059345 | Postrenal failure |
| 89 | 10059846 | Renal cyst haemorrhage |
| 90 | 10061481 | Renal injury |
| 91 | 10061835 | Diabetic nephropathy |
| 92 | 10061927 | Pyelocaliectasis |
| 93 | 10061989 | Glomerulosclerosis |
| 94 | 10062104 | Renal mass |
| 95 | 10062237 | Renal impairment |
| 96 | 10062550 | Renal salt-wasting syndrome |
| 97 | 10062553 | Scleroderma renal crisis |
| 98 | 10062622 | Pigment nephropathy |
| 99 | 10062854 | Renal hydrocele |
| 100 | 10063530 | Single functional kidney |
| 101 | 10063544 | Renal embolism |
| 102 | 10063897 | Renal ischaemia |
| 103 | 10064848 | Chronic kidney disease |
| 104 | 10065427 | Reflux nephropathy |
| 105 | 10065561 | Renal artery arteriosclerosis |
| 106 | 10065673 | Nephritic syndrome |
| 107 | 10065792 | Kidney perforation |
| 108 | 10066453 | Mesangioproliferative glomerulonephritis |
| 109 | 10067757 | Focal segmental glomerulosclerosis |
| 110 | 10067871 | Immunotactoid glomerulonephritis |
| 111 | 10068279 | Fibrillary glomerulonephritis |
| 112 | 10068513 | Pulmonary renal syndrome |
| 113 | 10069034 | Tubulointerstitial nephritis and uveitis syndrome |
| 114 | 10069339 | Acute kidney injury |
| 115 | 10069384 | Ischaemic nephropathy |
| 116 | 10069440 | Henoch-Schonlein purpura nephritis |
| 117 | 10069688 | Acute phosphate nephropathy |
| 118 | 10070869 | Acquired cystic kidney disease |
| 119 | 10071138 | Malnutrition-inflammation-atherosclerosis syndrome |
| 120 | 10071503 | Crystal nephropathy |
| 121 | 10072226 | Renal vascular thrombosis |
| 122 | 10072370 | Prerenal failure |
| 123 | 10073016 | Chronic autoimmune glomerulonephritis |
| 124 | 10073381 | Oedematous kidney |
| 125 | 10073599 | Myeloma cast nephropathy |

| | | |
|---|---|---|
| 126 | 10073745 | Hydrocalyx |
| 127 | 10074386 | Renal vein compression |
| 128 | 10074480 | Post infection glomerulonephritis |
| 129 | 10075626 | Paraneoplastic nephrotic syndrome |
| 130 | 10075737 | Renal artery perforation |
| 131 | 10075849 | Potassium wasting nephropathy |
| 132 | 10076704 | Page kidney |
| 133 | 10076749 | Paraneoplastic glomerulonephritis |
| 134 | 10077087 | Autoimmune nephritis |
| 135 | 10077209 | IgM nephropathy |
| 136 | 10077278 | Renal vein varices |
| 137 | 10077512 | End stage renal disease |
| 138 | 10077827 | C3 glomerulopathy |
| 139 | 10077862 | Obstructive nephropathy |
| 140 | 10078656 | Renal cell dysplasia |
| 141 | 10078907 | Renal vein stenosis |
| 142 | 10078933 | Renal tubular injury |
| 143 | 10078987 | Foetal renal impairment |
| 144 | 10080015 | Malignant urinary tract obstruction |
| 145 | 10081079 | Renal pseudoaneurysm |
| 146 | 10081461 | C1q nephropathy |
| 147 | 10081588 | Metabolic nephropathy |
| 148 | 10081980 | Subacute kidney injury |
| 149 | 10081981 | Anti-glomerular basement membrane disease |
| 150 | 10083070 | Immune-mediated nephritis |
| 151 | 10083098 | Membranous-like glomerulopathy with masked IgG-kappa deposits |
| 152 | 10083346 | Anticoagulant-related nephropathy |
| 153 | 10083385 | Subcapsular renal haematoma |
| 154 | 10083522 | Immune-mediated renal disorder |
| 155 | 10084204 | Sickle cell nephropathy |
| 156 | 10084543 | Renal phospholipidosis |
| 157 | 10084971 | Mesangiolipidosis |
| 158 | 10085346 | Renal-limited thrombotic microangiopathy |
| 159 | 10085438 | Anti-LRP2 nephropathy |
| 160 | 10086569 | Renal milk of calcium cyst |
| 161 | 10086674 | Balkan endemic nephropathy |
| 162 | 10086701 | Diabetic complication renal |
| 163 | 10087686 | APOL1-mediated kidney disease |

HLGT, High Level Group Term of MedDRA

**Supplementary Table S5**

Two-by-two contingency table for reporting odds ratio analysis.

| Drugs | CDI event cases | All other adverse event cases |
|---|---|---|
| Proton pump inhibitors | a | b |
| All other drugs | c | d |
| ROR = $\frac{a/b}{c/d}$, 95%CI for ROR = exp (ln (ROR) ± 1.96$\sqrt{\frac{1}{a}+\frac{1}{b}+\frac{1}{c}+\frac{1}{d}}$) | | |
| IC = $\log_2\frac{a(a+b+c+d)}{(a+b)(a+c)}$<br>E(IC) = $\log_2\frac{(a+\gamma11)(N+\alpha)(N+\beta)}{(N+\gamma)(a+b+\alpha1)(a+c+\beta1)}$<br>V(IC) ≈ $(\frac{1}{\log 2})^2[\frac{N-a+\gamma-\gamma11)}{(a+\gamma11)(1+N+\gamma)}+\frac{N-a-b+\alpha-\alpha1)}{(a+b+\alpha1)(1+N+\alpha)}+\frac{N-a-c+\beta-\beta1)}{(a+c+\beta1)(1+N+\beta)}]$<br>$\gamma = \gamma_{11}\frac{(N+\alpha)(N+\beta)}{(a+b+\alpha1)(a+c+\beta1)}$<br>95%CI for IC = E(IC) ± 1.96$\sqrt{V(IC)}$<br>Where $\alpha=\alpha_1+\alpha_2$, $\beta=\beta_1+\beta_2$, N=a+b+c+d, and the value of $\alpha_1$, $\alpha_2$, $\beta_1$, $\beta_2$ and $\gamma_{11}$ were defined as 1. | | |

CDI, Clostridioides difficile infection; ROR: reporting odds ratio; IC: information component; 95%CI: 95% confidence interval.

Supplementary Table S6

Disproportionality analysis of PPIs (primary or secondary suspect drug roles) and CDI.

| Analysis groups and Drugs | CDI event case | | ROR | | IC | |
|---|---|---|---|---|---|---|
| | number /n | proportion /% | ROR | 95%CI | IC | 95%CI |
| **No case Excluded*** | **1268** | **0.53%** | **2.82** | **2.67 to 2.98** | **1.46** | **1.27 to 1.64** |
| pantoprazole* | 400 | 0.80% | 4.16 | 3.77 to 4.60 | 2.04 | 1.70 to 2.35 |
| omeprazole* | 364 | 0.65% | 3.38 | 3.05 to 3.75 | 1.74 | 1.39 to 2.07 |
| lansoprazole* | 228 | 0.63% | 3.29 | 2.88 to 3.75 | 1.70 | 1.26 to 2.12 |
| rabeprazole* | 34 | 0.53% | 2.76 | 1.97 to 3.87 | 1.46 | 0.28 to 2.49 |
| dexlansoprazole* | 31 | 0.47% | 2.44 | 1.71 to 3.47 | 1.28 | 0.06 to 2.37 |
| esomeprazole | 206 | 0.25% | 1.29 | 1.13 to 1.48 | 0.37 | -0.09 to 0.82 |
| vonoprazan | 5 | 1.33% | 6.96 | 2.88 to 16.82 | 2.78 | -0.88 to 4.47 |
| **ISD cases Excluded*** | **1139** | **0.52%** | **2.74** | **2.58 to 2.91** | **1.42** | **1.22 to 1.61** |
| pantoprazole* | 326 | 0.76% | 3.98 | 3.57 to 4.44 | 1.98 | 1.60 to 2.33 |
| omeprazole* | 351 | 0.67% | 3.51 | 3.16 to 3.90 | 1.79 | 1.43 to 2.13 |
| lansoprazole* | 218 | 0.64% | 3.33 | 2.91 to 3.81 | 1.72 | 1.26 to 2.15 |
| rabeprazole* | 34 | 0.59% | 3.08 | 2.20 to 4.31 | 1.61 | 0.43 to 2.64 |
| dexlansoprazole | 25 | 0.41% | 2.11 | 1.42 to 3.12 | 1.07 | -0.27 to 2.29 |
| esomeprazole | 180 | 0.23% | 1.18 | 1.02 to 1.37 | 0.24 | -0.25 to 0.73 |
| vonoprazan | 5 | 1.49% | 7.80 | 3.23 to 18.87 | 2.94 | -0.82 to 4.54 |
| **ABD cases Excluded*** | **573** | **0.29%** | **1.51** | **1.39 to 1.64** | **0.58** | **0.30 to 0.85** |
| pantoprazole* | 184 | 0.47% | 2.42 | 2.09 to 2.80 | 1.27 | 0.77 to 1.74 |
| rabeprazole | 23 | 0.42% | 2.19 | 1.45 to 3.30 | 1.13 | -0.27 to 2.39 |
| dexlansoprazole | 24 | 0.41% | 2.12 | 1.42 to 3.17 | 1.08 | -0.29 to 2.32 |
| omeprazole* | 139 | 0.29% | 1.52 | 1.28 to 1.79 | 0.60 | 0.04 to 1.14 |
| lansoprazole | 68 | 0.23% | 1.18 | 0.93 to 1.50 | 0.24 | -0.55 to 1.02 |
| esomeprazole | 133 | 0.19% | 0.99 | 0.83 to 1.17 | -0.02 | -0.59 to 0.54 |
| vonoprazan | 2 | 0.88% | 4.58 | 1.14 to 18.41 | 2.18 | -2.73 to 4.84 |
| **ABD/ISD cases Excluded*** | **466** | **0.25%** | **1.30** | **1.19 to 1.43** | **0.37** | **0.07 to 0.68** |
| rabeprazole | 23 | 0.47% | 2.42 | 1.61 to 3.65 | 1.27 | -0.14 to 2.52 |
| dexlansoprazole | 21 | 0.38% | 1.98 | 1.29 to 3.04 | 0.98 | -0.47 to 2.31 |
| pantoprazole* | 119 | 0.35% | 1.79 | 1.50 to 2.15 | 0.84 | 0.23 to 1.42 |
| omeprazole | 126 | 0.28% | 1.46 | 1.22 to 1.73 | 0.54 | -0.05 to 1.11 |
| lansoprazole | 61 | 0.21% | 1.11 | 0.86 to 1.43 | 0.15 | -0.68 to 0.97 |
| esomeprazole | 114 | 0.17% | 0.87 | 0.73 to 1.05 | -0.20 | -0.80 to 0.42 |
| vonoprazan | 2 | 0.98% | 5.10 | 1.27 to 20.53 | 2.34 | -2.68 to 4.89 |
| **RI event cases Excluded*** | **1069** | **0.61%** | **3.23** | **3.04 to 3.44** | **1.65** | **1.45 to 1.85** |
| lansoprazole* | 199 | 0.98% | 5.11 | 4.44 to 5.87 | 2.33 | 1.84 to 2.77 |
| pantoprazole* | 349 | 0.92% | 4.81 | 4.33 to 5.35 | 2.24 | 1.88 to 2.58 |
| omeprazole* | 335 | 0.70% | 3.67 | 3.30 to 4.09 | 1.86 | 1.49 to 2.21 |
| dexlansoprazole | 19 | 0.50% | 2.60 | 1.65 to 4.08 | 1.37 | -0.20 to 2.72 |
| rabeprazole | 20 | 0.39% | 2.00 | 1.29 to 3.10 | 0.99 | -0.50 to 2.35 |

| | | | | | | |
|---|---|---|---|---|---|---|
| esomeprazole | 143 | 0.24% | 1.23 | 1.05 to 1.45 | 0.30 | -0.25 to 0.84 |
| vonoprazan | 4 | 1.16% | 6.05 | 2.26 to 16.22 | 2.58 | -1.35 to 4.52 |
| **ABD/ISD and RI event cases Excluded*** | **360** | **0.26%** | **1.34** | **1.2 to 1.48** | **0.41** | **0.07 to 0.76** |
| pantoprazole* | 99 | 0.39% | 2.00 | 1.64 to 2.44 | 1.00 | 0.33 to 1.64 |
| dexlansoprazole | 11 | 0.33% | 1.72 | 0.95 to 3.11 | 0.78 | -1.19 to 2.58 |
| rabeprazole | 12 | 0.31% | 1.63 | 0.92 to 2.87 | 0.70 | -1.18 to 2.44 |
| omeprazole* | 115 | 0.30% | 1.53 | 1.27 to 1.84 | 0.61 | 0.00 to 1.21 |
| lansoprazole | 41 | 0.27% | 1.40 | 1.03 to 1.90 | 0.48 | -0.54 to 1.47 |
| esomeprazole | 81 | 0.15% | 0.79 | 0.64 to 0.99 | -0.33 | -1.05 to 0.39 |
| vonoprazan | 1 | 0.55% | 2.83 | 0.4 to 20.19 | 1.50 | -4.07 to 5.19 |

CDI, Clostridioides difficile infection; ROR, reporting odds ratio; 95% CI, 95% confidence interval; IC, information component; ISD, immunosuppressive drugs; ABD, antibacterial drugs; RI, renal injury; *, significant dementia disproportionality signal detected.

Supplementary Table S7

Disproportionality analysis of PPIs (primary suspect drug role) and CDI based on different analyses and age groups.

| Analysis groups | CDI event case | | ROR | | IC | |
|---|---|---|---|---|---|---|
| | number /n | proportion /% | ROR | 95%CI | IC | 95%CI |
| **No case Excluded** | | | | | | |
| **18 years below** | 13 | 0.51 | 1.67 | 0.97 to 2.89 | 0.74 | -1.08 to 2.42 |
| lansoprazole | 5 | 1.19 | 3.97 | 1.64 to 9.59 | 1.97 | -1.28 to 4.08 |
| omeprazole | 5 | 0.43 | 1.44 | 0.60 to 3.46 | 0.52 | -2.25 to 3.09 |
| esomeprazole | 2 | 0.31 | 1.01 | 0.25 to 4.07 | 0.02 | -3.76 to 3.78 |
| pantoprazole | 1 | 0.32 | 1.05 | 0.15 to 7.48 | 0.07 | -4.59 to 4.65 |
| **18 to 64 years*** | 239 | 0.42 | 1.97 | 1.74 to 2.24 | 0.96 | 0.53 to 1.38 |
| Omeprazole* | 78 | 0.61 | 2.86 | 2.29 to 3.58 | 1.51 | 0.74 to 2.21 |
| Pantoprazole* | 73 | 0.70 | 3.30 | 2.62 to 4.15 | 1.71 | 0.90 to 2.43 |
| esomeprazole | 55 | 0.20 | 0.94 | 0.72 to 1.23 | -0.08 | -0.95 to 0.79 |
| lansoprazole | 21 | 0.46 | 2.13 | 1.39 to 3.27 | 1.09 | -0.38 to 2.41 |
| Dexlansoprazole* | 11 | 1.23 | 5.80 | 3.20 to 10.52 | 2.52 | 0.15 to 3.93 |
| rabeprazole | 1 | 0.08 | 0.39 | 0.05 to 2.76 | -1.36 | -5.44 to 3.77 |
| **65 years and above*** | 322 | 0.75 | 2.53 | 2.27 to 2.83 | 1.31 | 0.93 to 1.67 |
| Pantoprazole* | 123 | 1.45 | 4.87 | 4.08 to 5.83 | 2.26 | 1.62 to 2.80 |
| Omeprazole* | 78 | 0.68 | 2.25 | 1.80 to 2.82 | 1.16 | 0.40 to 1.88 |
| esomeprazole | 57 | 0.36 | 1.19 | 0.91 to 1.54 | 0.24 | -0.62 to 1.10 |
| Lansoprazole* | 53 | 1.01 | 3.38 | 2.58 to 4.44 | 1.74 | 0.79 to 2.57 |
| rabeprazole | 10 | 0.87 | 2.89 | 1.55 to 5.39 | 1.52 | -0.68 to 3.27 |
| dexlansoprazole | 1 | 0.16 | 0.52 | 0.07 to 3.73 | -0.93 | -5.15 to 4.07 |
| **unknown age*** | 197 | 0.29 | 2.51 | 2.18 to 2.89 | 1.31 | 0.83 to 1.76 |
| Pantoprazole* | 48 | 0.46 | 4.05 | 3.05 to 5.39 | 2.01 | 0.99 to 2.86 |
| Lansoprazole* | 45 | 0.27 | 2.39 | 1.78 to 3.20 | 1.25 | 0.24 to 2.17 |
| esomeprazole | 42 | 0.17 | 1.49 | 1.10 to 2.01 | 0.57 | -0.44 to 1.55 |
| Omeprazole* | 32 | 0.26 | 2.30 | 1.62 to 3.25 | 1.19 | 0.00 to 2.28 |
| Rabeprazole* | 15 | 1.02 | 8.90 | 5.35 to 14.81 | 3.14 | 0.93 to 4.20 |
| dexlansoprazole | 14 | 0.34 | 2.91 | 1.72 to 4.92 | 1.54 | -0.32 to 3.05 |
| vonoprazan | 1 | 7.69 | 72.02 | 9.36 to 553.93 | 6.06 | -3.95 to 5.90 |
| **ISD case Excluded** | | | | | | |
| **18 years below** | 12 | 0.50 | 1.65 | 0.94 to 2.92 | 0.72 | -1.17 to 2.47 |
| lansoprazole | 5 | 1.22 | 4.08 | 1.69 to 9.86 | 2.01 | -1.26 to 4.10 |
| omeprazole | 5 | 0.47 | 1.56 | 0.65 to 3.77 | 0.64 | -2.16 to 3.18 |
| esomeprazole | 2 | 0.32 | 1.06 | 0.26 to 4.25 | 0.08 | -3.72 to 3.82 |
| **18 to 64 years*** | 215 | 0.40 | 1.88 | 1.64 to 2.15 | 0.90 | 0.44 to 1.34 |
| Omeprazole* | 78 | 0.64 | 3.01 | 2.41 to 3.76 | 1.58 | 0.80 to 2.28 |
| Pantoprazole* | 61 | 0.66 | 3.12 | 2.42 to 4.01 | 1.63 | 0.75 to 2.41 |

| | | | | | | |
|---|---|---|---|---|---|---|
| esomeprazole | 47 | 0.18 | 0.84 | 0.63 to 1.12 | -0.25 | -1.19 to 0.69 |
| lansoprazole | 18 | 0.41 | 1.93 | 1.21 to 3.06 | 0.94 | -0.63 to 2.37 |
| dexlansoprazole | 10 | 1.16 | 5.44 | 2.92 to 10.16 | 2.43 | -0.03 to 3.92 |
| rabeprazole | 1 | 0.09 | 0.42 | 0.06 to 2.96 | -1.26 | -5.37 to 3.84 |
| **65 years and above*** | 308 | 0.75 | 2.53 | 2.26 to 2.83 | 1.31 | 0.92 to 1.68 |
| Pantoprazole* | 114 | 1.44 | 4.85 | 4.03 to 5.85 | 2.25 | 1.59 to 2.82 |
| Omeprazole* | 78 | 0.71 | 2.35 | 1.88 to 2.94 | 1.22 | 0.46 to 1.94 |
| esomeprazole | 54 | 0.35 | 1.16 | 0.89 to 1.52 | 0.21 | -0.67 to 1.09 |
| Lansoprazole* | 51 | 1.01 | 3.37 | 2.55 to 4.44 | 1.74 | 0.76 to 2.58 |
| rabeprazole | 10 | 0.90 | 3.00 | 1.61 to 5.59 | 1.58 | -0.64 to 3.31 |
| dexlansoprazole | 1 | 0.16 | 0.54 | 0.08 to 3.85 | -0.88 | -5.12 to 4.10 |
| **unknown age*** | 193 | 0.28 | 2.50 | 2.17 to 2.88 | 1.30 | 0.82 to 1.76 |
| Pantoprazole* | 46 | 0.46 | 4.01 | 3.00 to 5.36 | 1.99 | 0.95 to 2.86 |
| Lansoprazole* | 44 | 0.27 | 2.37 | 1.76 to 3.19 | 1.24 | 0.22 to 2.17 |
| esomeprazole | 42 | 0.17 | 1.50 | 1.11 to 2.03 | 0.58 | -0.43 to 1.56 |
| omeprazole | 31 | 0.26 | 2.28 | 1.60 to 3.24 | 1.18 | -0.03 to 2.28 |
| Rabeprazole* | 15 | 1.03 | 9.05 | 5.44 to 15.05 | 3.16 | 0.94 to 4.22 |
| dexlansoprazole | 14 | 0.34 | 2.94 | 1.74 to 4.98 | 1.55 | -0.31 to 3.06 |
| vonoprazan | 1 | 8.33 | 78.56 | 10.14 to 608.60 | 6.17 | -3.97 to 5.92 |
| **ABD case Excluded** | | | | | | |
| **18 years below** | 12 | 0.54 | 1.80 | 1.02 to 3.18 | 0.84 | -1.06 to 2.57 |
| omeprazole | 5 | 0.52 | 1.71 | 0.71 to 4.11 | 0.77 | -2.07 to 3.28 |
| lansoprazole | 4 | 1.05 | 3.50 | 1.31 to 9.39 | 1.79 | -1.72 to 4.15 |
| esomeprazole | 2 | 0.34 | 1.14 | 0.28 to 4.55 | 0.18 | -3.66 to 3.89 |
| pantoprazole | 1 | 0.41 | 1.35 | 0.19 to 9.61 | 0.43 | -4.43 to 4.82 |
| **18 to 64 years** | 124 | 0.27 | 1.25 | 1.05 to 1.49 | 0.32 | -0.27 to 0.90 |
| esomeprazole | 41 | 0.19 | 0.87 | 0.64 to 1.19 | -0.19 | -1.20 to 0.82 |
| pantoprazole | 37 | 0.46 | 2.15 | 1.55 to 2.97 | 1.10 | -0.01 to 2.11 |
| omeprazole | 29 | 0.27 | 1.27 | 0.88 to 1.83 | 0.34 | -0.86 to 1.52 |
| dexlansoprazole | 8 | 0.97 | 4.54 | 2.26 to 9.10 | 2.17 | -0.49 to 3.88 |
| lansoprazole | 8 | 0.21 | 0.96 | 0.48 to 1.92 | -0.06 | -2.23 to 2.12 |
| rabeprazole | 1 | 0.11 | 0.49 | 0.07 to 3.50 | -1.02 | -5.21 to 4.01 |
| **65 years and above** | 134 | 0.37 | 1.23 | 1.03 to 1.46 | 0.29 | -0.28 to 0.85 |
| Pantoprazole* | 45 | 0.65 | 2.16 | 1.61 to 2.90 | 1.10 | 0.10 to 2.03 |
| omeprazole | 35 | 0.35 | 1.14 | 0.82 to 1.60 | 0.19 | -0.90 to 1.28 |
| esomeprazole | 33 | 0.25 | 0.83 | 0.59 to 1.16 | -0.27 | -1.39 to 0.85 |
| lansoprazole | 15 | 0.34 | 1.13 | 0.68 to 1.87 | 0.17 | -1.47 to 1.79 |
| rabeprazole | 5 | 0.51 | 1.69 | 0.70 to 4.08 | 0.76 | -2.07 to 3.27 |
| dexlansoprazole | 1 | 0.17 | 0.55 | 0.08 to 3.92 | -0.86 | -5.11 to 4.12 |
| **unknown age*** | 152 | 0.24 | 2.07 | 1.77 to 2.43 | 1.04 | 0.49 to 1.56 |
| esomeprazole | 36 | 0.15 | 1.34 | 0.96 to 1.85 | 0.42 | -0.67 to 1.48 |
| Pantoprazole* | 34 | 0.35 | 3.08 | 2.20 to 4.32 | 1.62 | 0.43 to 2.64 |
| lansoprazole | 30 | 0.21 | 1.79 | 1.25 to 2.56 | 0.83 | -0.38 to 1.97 |
| omeprazole | 24 | 0.21 | 1.82 | 1.22 to 2.71 | 0.86 | -0.49 to 2.12 |

| | | | | | | |
|---|---|---|---|---|---|---|
| Rabeprazole* | 15 | 1.05 | 9.15 | 5.50 to 15.23 | 3.18 | 0.95 to 4.23 |
| dexlansoprazole | 12 | 0.32 | 2.81 | 1.59 to 4.95 | 1.48 | -0.51 to 3.11 |
| vonoprazan | 1 | 9.09 | 86.42 | 11.06 to 675.18 | 6.30 | -3.99 to 5.95 |
| **ABD/ISD case Excluded** | | | | | | |
| **18 years below** | 11 | 0.52 | 1.73 | 0.96 to 3.14 | 0.79 | -1.19 to 2.59 |
| omeprazole | 5 | 0.55 | 1.82 | 0.76 to 4.39 | 0.86 | -2.00 to 3.35 |
| lansoprazole | 4 | 1.06 | 3.52 | 1.31 to 9.44 | 1.80 | -1.72 to 4.15 |
| esomeprazole | 2 | 0.36 | 1.17 | 0.29 to 4.70 | 0.23 | -3.63 to 3.92 |
| **18 to 64 years** | 105 | 0.24 | 1.11 | 0.92 to 1.35 | 0.15 | -0.49 to 0.78 |
| esomeprazole | 33 | 0.16 | 0.72 | 0.51 to 1.01 | -0.47 | -1.58 to 0.66 |
| omeprazole | 29 | 0.28 | 1.32 | 0.92 to 1.90 | 0.40 | -0.81 to 1.58 |
| pantoprazole | 26 | 0.37 | 1.71 | 1.16 to 2.51 | 0.77 | -0.53 to 1.99 |
| dexlansoprazole | 8 | 1.00 | 4.67 | 2.32 to 9.37 | 2.21 | -0.46 to 3.90 |
| lansoprazole | 8 | 0.21 | 1.00 | 0.50 to 2.00 | -0.00 | -2.18 to 2.17 |
| rabeprazole | 1 | 0.11 | 0.53 | 0.07 to 3.76 | -0.92 | -5.14 to 4.08 |
| **65 years and above** | 125 | 0.36 | 1.19 | 0.99 to 1.42 | 0.24 | -0.34 to 0.83 |
| pantoprazole | 38 | 0.59 | 1.96 | 1.43 to 2.70 | 0.97 | -0.12 to 1.98 |
| omeprazole | 35 | 0.36 | 1.19 | 0.85 to 1.66 | 0.25 | -0.85 to 1.33 |
| esomeprazole | 33 | 0.26 | 0.84 | 0.60 to 1.19 | -0.24 | -1.36 to 0.88 |
| lansoprazole | 13 | 0.30 | 1.00 | 0.58 to 1.73 | 0.00 | -1.74 to 1.75 |
| rabeprazole | 5 | 0.53 | 1.76 | 0.73 to 4.23 | 0.81 | -2.03 to 3.30 |
| dexlansoprazole | 1 | 0.17 | 0.57 | 0.08 to 4.04 | -0.81 | -5.08 to 4.15 |
| **unknown age*** | 149 | 0.24 | 2.06 | 1.75 to 2.42 | 1.03 | 0.48 to 1.55 |
| esomeprazole* | 36 | 0.16 | 1.35 | 0.97 to 1.87 | 0.43 | -0.66 to 1.49 |
| Pantoprazole* | 33 | 0.35 | 3.07 | 2.18 to 4.33 | 1.61 | 0.41 to 2.65 |
| lansoprazole | 29 | 0.20 | 1.74 | 1.21 to 2.51 | 0.80 | -0.43 to 1.95 |
| omeprazole | 23 | 0.20 | 1.77 | 1.18 to 2.67 | 0.82 | -0.56 to 2.11 |
| Rabeprazole* | 15 | 1.06 | 9.26 | 5.56 to 15.41 | 3.19 | 0.96 to 4.24 |
| dexlansoprazole | 12 | 0.33 | 2.83 | 1.60 to 4.99 | 1.50 | -0.50 to 3.12 |
| vonoprazan | 1 | 10.00 | 96.02 | 12.16 to 758.01 | 6.43 | -4.02 to 5.99 |
| **ABD/ISD and RI event case Excluded** | | | | | | |
| **18 years below** | 11 | 0.54 | 1.78 | 0.98 to 3.23 | 0.83 | -1.16 to 2.63 |
| omeprazole | 5 | 0.56 | 1.86 | 0.77 to 4.49 | 0.89 | -1.97 to 3.37 |
| lansoprazole | 4 | 1.10 | 3.65 | 1.36 to 9.78 | 1.85 | -1.69 to 4.18 |
| esomeprazole | 2 | 0.36 | 1.20 | 0.30 to 4.82 | 0.26 | -3.60 to 3.94 |
| **18 to 64 years** | 90 | 0.25 | 1.15 | 0.93 to 1.42 | 0.20 | -0.49 to 0.88 |
| omeprazole | 26 | 0.29 | 1.35 | 0.92 to 1.98 | 0.43 | -0.85 to 1.67 |
| esomeprazole | 24 | 0.13 | 0.62 | 0.42 to 0.93 | -0.68 | -1.96 to 0.65 |
| pantoprazole | 24 | 0.47 | 2.21 | 1.48 to 3.30 | 1.14 | -0.24 to 2.38 |
| dexlansoprazole | 8 | 1.13 | 5.31 | 2.64 to 10.65 | 2.39 | -0.35 to 4.02 |
| lansoprazole | 7 | 0.23 | 1.09 | 0.52 to 2.28 | 0.12 | -2.20 to 2.41 |
| rabeprazole | 1 | 0.12 | 0.56 | 0.08 to 4.00 | -0.83 | -5.08 to 4.14 |
| **65 years and above** | 109 | 0.36 | 1.20 | 0.99 to 1.45 | 0.26 | -0.37 to 0.88 |
| pantoprazole | 34 | 0.65 | 2.16 | 1.54 to 3.02 | 1.10 | -0.05 to 2.16 |

| | | | | | | |
|---|---|---|---|---|---|---|
| omeprazole | 32 | 0.38 | 1.26 | 0.89 to 1.78 | 0.33 | -0.82 to 1.45 |
| esomeprazole | 27 | 0.24 | 0.80 | 0.55 to 1.16 | -0.33 | -1.55 to 0.92 |
| lansoprazole | 11 | 0.29 | 0.95 | 0.53 to 1.73 | -0.07 | -1.95 to 1.82 |
| rabeprazole | 5 | 0.57 | 1.90 | 0.79 to 4.58 | 0.92 | -1.95 to 3.39 |
| **unknown age*** | 81 | 0.24 | 2.12 | 1.70 to 2.64 | 1.08 | 0.33 to 1.78 |
| Pantoprazole* | 23 | 0.50 | 4.35 | 2.88 to 6.55 | 2.11 | 0.59 to 3.26 |
| omeprazole | 20 | 0.22 | 1.93 | 1.25 to 3.00 | 0.95 | -0.54 to 2.31 |
| esomeprazole | 18 | 0.12 | 1.07 | 0.67 to 1.70 | 0.10 | -1.40 to 1.59 |
| lansoprazole | 12 | 0.42 | 3.65 | 2.07 to 6.44 | 1.86 | -0.22 to 3.41 |
| rabeprazole | 4 | 0.67 | 5.87 | 2.20 to 15.70 | 2.54 | -1.36 to 4.49 |
| dexlansoprazole | 3 | 0.18 | 1.57 | 0.51 to 4.87 | 0.65 | -2.80 to 3.72 |
| vonoprazan | 1 | 11.11 | 108.02 | 13.51 to 863.81 | 6.59 | -4.06 to 6.03 |

CDI, Clostridioides difficile infection; ROR, reporting odds ratio; 95% CI, 95% confidence interval; IC, information component; ISD, immunosuppressive drugs; ABD, antibacterial drugs; RI, renal injury; *, significant dementia disproportionality signal detected.

Supplementary Table S8

Disproportionality analysis of PPIs (primary or secondary suspect drug roles) and CDI based on different analyses and age groups.

| Analysis groups | CDI event case | | ROR | | IC | |
|---|---|---|---|---|---|---|
| | number /n | proportion /% | ROR | 95%CI | IC | 95%CI |
| **No case Excluded** | | | | | | |
| **All ages** | 1268 | 0.53 | 2.82 | 2.67 to 2.98 | 1.46 | 1.27 to 1.64 |
| **18 years below** | 17 | 0.42 | 1.39 | 0.86 to 2.25 | 0.47 | -1.11 to 1.99 |
| lansoprazole | 6 | 0.88 | 2.94 | 1.31 to 6.58 | 1.54 | -1.29 to 3.67 |
| omeprazole | 6 | 0.34 | 1.13 | 0.51 to 2.53 | 0.18 | -2.32 to 2.62 |
| pantoprazole | 3 | 0.46 | 1.53 | 0.49 to 4.76 | 0.61 | -2.84 to 3.70 |
| esomeprazole | 2 | 0.22 | 0.74 | 0.18 to 2.95 | -0.44 | -4.08 to 3.46 |
| **18 to 64 yr PPIs*** | 388 | 0.47 | 2.25 | 2.03 to 2.48 | 1.14 | 0.80 to 1.47 |
| omeprazole* | 128 | 0.66 | 3.11 | 2.61 to 3.70 | 1.62 | 1.02 to 2.17 |
| pantoprazole* | 128 | 0.70 | 3.28 | 2.76 to 3.91 | 1.70 | 1.09 to 2.25 |
| esomeprazole | 69 | 0.21 | 0.97 | 0.77 to 1.23 | -0.04 | -0.82 to 0.74 |
| lansoprazole* | 44 | 0.58 | 2.69 | 2.00 to 3.63 | 1.42 | 0.39 to 2.34 |
| dexlansoprazole* | 15 | 1.15 | 5.39 | 3.24 to 8.97 | 2.41 | 0.43 to 3.71 |
| rabeprazole | 4 | 0.19 | 0.88 | 0.33 to 2.34 | -0.19 | -3.07 to 2.76 |
| **65 years and above*** | 589 | 0.85 | 2.92 | 2.68 to 3.17 | 1.48 | 1.20 to 1.75 |
| pantoprazole* | 189 | 1.17 | 3.94 | 3.41 to 4.55 | 1.95 | 1.44 to 2.40 |
| omeprazole* | 177 | 0.94 | 3.14 | 2.71 to 3.65 | 1.63 | 1.12 to 2.10 |
| lansoprazole* | 119 | 1.23 | 4.13 | 3.45 to 4.96 | 2.02 | 1.38 to 2.59 |
| esomeprazole | 84 | 0.39 | 1.30 | 1.05 to 1.61 | 0.37 | -0.34 to 1.08 |
| rabeprazole | 15 | 0.66 | 2.21 | 1.33 to 3.67 | 1.14 | -0.60 to 2.67 |
| vonoprazan | 4 | 2.00 | 6.72 | 2.5 to 18.09 | 2.72 | -1.31 to 4.58 |
| dexlansoprazole | 1 | 0.11 | 0.37 | 0.05 to 2.62 | -1.44 | -5.50 to 3.72 |
| **unknown age*** | 274 | 0.33 | 2.93 | 2.60 to 3.31 | 1.52 | 1.11 to 1.91 |
| pantoprazole* | 80 | 0.53 | 4.64 | 3.72 to 5.79 | 2.20 | 1.41 to 2.86 |
| lansoprazole* | 59 | 0.33 | 2.84 | 2.19 to 3.67 | 1.49 | 0.61 to 2.30 |
| omeprazole* | 53 | 0.33 | 2.84 | 2.17 to 3.73 | 1.50 | 0.56 to 2.34 |
| esomeprazole | 51 | 0.19 | 1.64 | 1.24 to 2.15 | 0.71 | -0.22 to 1.59 |
| dexlansoprazole | 15 | 0.34 | 2.98 | 1.79 to 4.95 | 1.57 | -0.23 to 3.04 |
| rabeprazole* | 15 | 0.77 | 6.71 | 4.04 to 11.16 | 2.74 | 0.66 to 3.93 |
| vonoprazan | 1 | 2.08 | 18.39 | 2.54 to 133.29 | 4.17 | -3.78 to 5.62 |
| **ISD case Excluded** | | | | | | |
| **All ages*** | 1139 | 0.52 | 2.74 | 2.58 to 2.91 | 1.42 | 1.22 to 1.61 |
| **18 years below** | 12 | 0.34 | 1.11 | 0.63 to 1.97 | 0.15 | -1.68 to 1.96 |
| lansoprazole | 5 | 0.80 | 2.65 | 1.10 to 6.40 | 1.40 | -1.63 to 3.72 |
| omeprazole | 5 | 0.33 | 1.10 | 0.46 to 2.65 | 0.14 | -2.56 to 2.78 |
| esomeprazole | 2 | 0.25 | 0.81 | 0.20 to 3.26 | -0.30 | -3.98 to 3.56 |

| | | | | | | |
|---|---|---|---|---|---|---|
| **18 to 64 yr PPIs*** | 318 | 0.44 | 2.06 | 1.84 to 2.30 | 1.02 | 0.64 to 1.38 |
| omeprazole* | 123 | 0.69 | 3.26 | 2.73 to 3.89 | 1.69 | 1.07 to 2.25 |
| pantoprazole* | 88 | 0.61 | 2.86 | 2.32 to 3.53 | 1.51 | 0.78 to 2.17 |
| esomeprazole | 52 | 0.17 | 0.78 | 0.59 to 1.02 | -0.36 | -1.25 to 0.54 |
| lansoprazole* | 41 | 0.60 | 2.81 | 2.07 to 3.82 | 1.48 | 0.41 to 2.43 |
| dexlansoprazole | 10 | 0.94 | 4.42 | 2.37 to 8.24 | 2.13 | -0.23 to 3.72 |
| rabeprazole | 4 | 0.22 | 1.05 | 0.39 to 2.79 | 0.06 | -2.87 to 2.97 |
| **65 years and above*** | 550 | 0.85 | 2.92 | 2.68 to 3.18 | 1.49 | 1.20 to 1.77 |
| omeprazole* | 175 | 0.99 | 3.33 | 2.86 to 3.87 | 1.71 | 1.19 to 2.19 |
| pantoprazole* | 163 | 1.13 | 3.79 | 3.24 to 4.43 | 1.89 | 1.36 to 2.39 |
| lansoprazole* | 114 | 1.26 | 4.25 | 3.53 to 5.12 | 2.06 | 1.41 to 2.64 |
| esomeprazole | 78 | 0.38 | 1.26 | 1.01 to 1.58 | 0.33 | -0.41 to 1.07 |
| rabeprazole | 15 | 0.72 | 2.40 | 1.44 to 3.99 | 1.25 | -0.5 to 2.77 |
| vonoprazan | 4 | 2.22 | 7.49 | 2.78 to 20.17 | 2.88 | -1.26 to 4.64 |
| dexlansoprazole | 1 | 0.12 | 0.41 | 0.06 to 2.93 | -1.28 | -5.39 to 3.83 |
| **unknown age*** | 259 | 0.33 | 2.91 | 2.57 to 3.30 | 1.51 | 1.09 to 1.91 |
| pantoprazole* | 75 | 0.56 | 4.93 | 3.92 to 6.19 | 2.28 | 1.46 to 2.96 |
| lansoprazole* | 58 | 0.33 | 2.87 | 2.21 to 3.71 | 1.51 | 0.61 to 2.32 |
| esomeprazole | 48 | 0.18 | 1.58 | 1.19 to 2.10 | 0.66 | -0.29 to 1.57 |
| omeprazole* | 48 | 0.31 | 2.73 | 2.06 to 3.63 | 1.44 | 0.46 to 2.33 |
| rabeprazole* | 15 | 0.83 | 7.22 | 4.34 to 12.00 | 2.84 | 0.73 to 4.00 |
| dexlansoprazole | 14 | 0.33 | 2.85 | 1.69 to 4.83 | 1.51 | -0.34 to 3.03 |
| vonoprazan | 1 | 2.33 | 20.58 | 2.83 to 149.52 | 4.33 | -3.78 to 5.64 |
| **ISD case Excluded** | | | | | | |
| **All ages*** | 573 | 0.29 | 1.51 | 1.39 to 1.64 | 0.58 | 0.30 to 0.85 |
| **18 years below** | 15 | 0.49 | 1.62 | 0.97 to 2.69 | 0.69 | -1.01 to 2.27 |
| omeprazole | 6 | 0.47 | 1.55 | 0.69 to 3.46 | 0.63 | -1.95 to 2.99 |
| lansoprazole | 5 | 0.97 | 3.23 | 1.34 to 7.80 | 1.68 | -1.45 to 3.90 |
| esomeprazole | 2 | 0.28 | 0.92 | 0.23 to 3.71 | -0.11 | -3.85 to 3.69 |
| pantoprazole | 2 | 0.40 | 1.33 | 0.33 to 5.34 | 0.41 | -3.52 to 4.03 |
| **18 to 64 yr PPIs** | 179 | 0.28 | 1.31 | 1.13 to 1.51 | 0.38 | -0.11 to 0.87 |
| pantoprazole* | 68 | 0.51 | 2.37 | 1.87 to 3.01 | 1.24 | 0.42 to 2.00 |
| esomeprazole | 51 | 0.20 | 0.91 | 0.69 to 1.19 | -0.14 | -1.04 to 0.77 |
| omeprazole | 41 | 0.26 | 1.22 | 0.89 to 1.65 | 0.28 | -0.73 to 1.28 |
| dexlansoprazole | 10 | 0.85 | 3.98 | 2.13 to 7.41 | 1.98 | -0.34 to 3.61 |
| lansoprazole | 8 | 0.14 | 0.63 | 0.32 to 1.27 | -0.66 | -2.77 to 1.58 |
| rabeprazole | 1 | 0.06 | 0.28 | 0.04 to 2.01 | -1.82 | -5.79 to 3.43 |
| **65 years and above** | 191 | 0.34 | 1.13 | 0.98 to 1.30 | 0.17 | -0.31 to 0.64 |
| pantoprazole | 66 | 0.53 | 1.74 | 1.37 to 2.22 | 0.79 | -0.02 to 1.58 |
| omeprazole | 56 | 0.35 | 1.17 | 0.90 to 1.53 | 0.23 | -0.64 to 1.09 |
| esomeprazole | 38 | 0.22 | 0.72 | 0.52 to 0.99 | -0.48 | -1.51 to 0.58 |
| lansoprazole | 22 | 0.29 | 0.97 | 0.64 to 1.48 | -0.04 | -1.40 to 1.32 |
| rabeprazole | 7 | 0.37 | 1.21 | 0.57 to 2.54 | 0.27 | -2.07 to 2.54 |
| dexlansoprazole | 1 | 0.13 | 0.42 | 0.06 to 3.01 | -1.24 | -5.36 to 3.86 |

| | | | | | | |
|---|---|---|---|---|---|---|
| vonoprazan | 1 | 0.75 | 2.48 | 0.35 to 17.71 | 1.30 | -4.13 to 5.14 |
| **unknown age*** | 188 | 0.25 | 2.21 | 1.92 to 2.56 | 1.13 | 0.64 to 1.60 |
| pantoprazole* | 48 | 0.37 | 3.23 | 2.43 to 4.29 | 1.68 | 0.68 to 2.55 |
| esomeprazole | 42 | 0.17 | 1.44 | 1.06 to 1.95 | 0.52 | -0.49 to 1.50 |
| omeprazole | 36 | 0.25 | 2.13 | 1.54 to 2.96 | 1.09 | -0.03 to 2.12 |
| lansoprazole | 33 | 0.21 | 1.81 | 1.29 to 2.55 | 0.85 | -0.30 to 1.94 |
| rabeprazole* | 15 | 0.83 | 7.22 | 4.34 to 12.00 | 2.84 | 0.73 to 4.00 |
| dexlansoprazole | 13 | 0.34 | 2.91 | 1.69 to 5.02 | 1.54 | -0.39 to 3.10 |
| vonoprazan | 1 | 2.63 | 23.36 | 3.2 to 170.25 | 4.51 | -3.78 to 5.66 |
| **ABD/ISD case Excluded** | | | | | | |
| **All ages*** | 466 | 0.25 | 1.30 | 1.19 to 1.43 | 0.37 | 0.07 to 0.68 |
| **18 years below** | 11 | 0.39 | 1.28 | 0.71 to 2.31 | 0.35 | -1.57 to 2.21 |
| omeprazole | 5 | 0.43 | 1.42 | 0.59 to 3.42 | 0.50 | -2.26 to 3.07 |
| lansoprazole | 4 | 0.80 | 2.64 | 0.99 to 7.08 | 1.39 | -1.95 to 3.91 |
| esomeprazole | 2 | 0.30 | 0.98 | 0.24 to 3.93 | -0.03 | -3.79 to 3.75 |
| **18 to 64 yr PPIs** | 120 | 0.21 | 0.96 | 0.80 to 1.15 | -0.06 | -0.65 to 0.54 |
| omeprazole | 36 | 0.25 | 1.15 | 0.83 to 1.59 | 0.20 | -0.88 to 1.26 |
| esomeprazole | 35 | 0.14 | 0.65 | 0.47 to 0.91 | -0.62 | -1.69 to 0.48 |
| pantoprazole | 32 | 0.30 | 1.39 | 0.98 to 1.96 | 0.47 | -0.68 to 1.59 |
| dexlansoprazole | 8 | 0.81 | 3.80 | 1.89 to 7.62 | 1.92 | -0.65 to 3.71 |
| lansoprazole | 8 | 0.15 | 0.70 | 0.35 to 1.40 | -0.51 | -2.64 to 1.71 |
| rabeprazole | 1 | 0.07 | 0.34 | 0.05 to 2.42 | -1.55 | -5.58 to 3.63 |
| **65 years and above** | 161 | 0.31 | 1.01 | 0.86 to 1.18 | 0.01 | -0.50 to 0.53 |
| omeprazole | 54 | 0.36 | 1.19 | 0.91 to 1.56 | 0.25 | -0.63 to 1.13 |
| pantoprazole | 43 | 0.38 | 1.26 | 0.94 to 1.71 | 0.34 | -0.66 to 1.31 |
| esomeprazole | 38 | 0.23 | 0.74 | 0.54 to 1.02 | -0.42 | -1.46 to 0.63 |
| lansoprazole | 17 | 0.24 | 0.79 | 0.49 to 1.28 | -0.33 | -1.85 to 1.22 |
| rabeprazole | 7 | 0.40 | 1.32 | 0.63 to 2.77 | 0.39 | -1.97 to 2.65 |
| dexlansoprazole | 1 | 0.14 | 0.48 | 0.07 to 3.39 | -1.06 | -5.24 to 3.98 |
| vonoprazan | 1 | 0.79 | 2.63 | 0.37 to 18.85 | 1.39 | -4.11 to 5.17 |
| **unknown age*** | 174 | 0.24 | 2.12 | 1.82 to 2.46 | 1.07 | 0.56 to 1.56 |
| pantoprazole* | 44 | 0.37 | 3.22 | 2.39 to 4.33 | 1.68 | 0.63 to 2.58 |
| esomeprazole | 39 | 0.16 | 1.36 | 0.99 to 1.86 | 0.44 | -0.61 to 1.46 |
| lansoprazole | 32 | 0.21 | 1.79 | 1.26 to 2.53 | 0.84 | -0.34 to 1.94 |
| omeprazole | 31 | 0.22 | 1.92 | 1.35 to 2.73 | 0.94 | -0.26 to 2.05 |
| rabeprazole* | 15 | 0.86 | 7.55 | 4.54 to 12.55 | 2.90 | 0.77 to 4.05 |
| dexlansoprazole | 12 | 0.32 | 2.75 | 1.56 to 4.84 | 1.45 | -0.54 to 3.08 |
| vonoprazan | 1 | 2.94 | 26.19 | 3.58 to 191.5 | 4.67 | -3.79 to 5.68 |
| **ABD/ISD and RI event case Excluded** | | | | | | |
| **All ages*** | 360 | 0.26 | 1.34 | 1.2 to 1.48 | 0.41 | 0.07 to 0.76 |
| **18 years below** | 11 | 0.40 | 1.32 | 0.73 to 2.4 | 0.40 | -1.53 to 2.25 |
| omeprazole | 5 | 0.45 | 1.48 | 0.61 to 3.56 | 0.56 | -2.22 to 3.12 |
| lansoprazole | 4 | 0.82 | 2.72 | 1.02 to 7.28 | 1.43 | -1.92 to 3.94 |

| | | | | | | |
|---|---|---|---|---|---|---|
| esomeprazole | 2 | 0.31 | 1.01 | 0.25 to 4.06 | 0.02 | -3.76 to 3.78 |
| **18 to 64 yr PPIs** | 102 | 0.21 | 0.96 | 0.79 to 1.17 | -0.06 | -0.70 to 0.59 |
| omeprazole | 32 | 0.24 | 1.14 | 0.81 to 1.61 | 0.19 | -0.95 to 1.32 |
| pantoprazole | 28 | 0.33 | 1.53 | 1.05 to 2.22 | 0.61 | -0.63 to 1.80 |
| esomeprazole | 26 | 0.12 | 0.57 | 0.39 to 0.84 | -0.81 | -2.04 to 0.47 |
| dexlansoprazole | 8 | 0.90 | 4.22 | 2.10 to 8.47 | 2.07 | -0.55 to 3.81 |
| lansoprazole | 7 | 0.16 | 0.73 | 0.35 to 1.53 | -0.45 | -2.71 to 1.90 |
| rabeprazole | 1 | 0.08 | 0.36 | 0.05 to 2.58 | -1.46 | -5.52 to 3.70 |
| **65 years and above** | 142 | 0.31 | 1.02 | 0.86 to 1.20 | 0.02 | -0.53 to 0.57 |
| omeprazole | 50 | 0.38 | 1.26 | 0.95 to 1.66 | 0.33 | -0.60 to 1.23 |
| pantoprazole | 38 | 0.39 | 1.30 | 0.95 to 1.79 | 0.38 | -0.68 to 1.42 |
| esomeprazole | 32 | 0.22 | 0.72 | 0.51 to 1.02 | -0.48 | -1.60 to 0.67 |
| lansoprazole | 15 | 0.24 | 0.78 | 0.47 to 1.29 | -0.36 | -1.97 to 1.29 |
| rabeprazole | 7 | 0.44 | 1.45 | 0.69 to 3.05 | 0.53 | -1.85 to 2.76 |
| **unknown age*** | 105 | 0.26 | 2.22 | 1.83 to 2.70 | 1.14 | 0.49 to 1.76 |
| pantoprazole* | 33 | 0.47 | 4.10 | 2.91 to 5.78 | 2.03 | 0.78 to 3.02 |
| omeprazole | 28 | 0.24 | 2.10 | 1.45 to 3.05 | 1.07 | -0.20 to 2.23 |
| esomeprazole | 21 | 0.13 | 1.13 | 0.74 to 1.74 | 0.18 | -1.22 to 1.56 |
| lansoprazole | 15 | 0.39 | 3.36 | 2.03 to 5.59 | 1.74 | -0.09 to 3.18 |
| rabeprazole | 4 | 0.45 | 3.93 | 1.47 to 10.49 | 1.97 | -1.62 to 4.23 |
| dexlansoprazole | 3 | 0.17 | 1.47 | 0.47 to 4.57 | 0.56 | -2.86 to 3.66 |
| vonoprazan | 1 | 3.45 | 30.86 | 4.2 to 226.87 | 4.90 | -3.80 to 5.71 |

CDI, Clostridioides difficile infection; ROR, reporting odds ratio; 95% CI, 95% confidence interval; IC, information component; ISD, immunosuppressive drugs; ABD, antibacterial drugs; RI, renal injury; *, significant dementia disproportionality signal detected.

**Supplementary Figure S1**

The reported trend of PPI cases in the FAERS database from 2004 to 2023.

Figure A, case identified by primary suspect drug role; Figure B, case identified by primary and secondary suspect drug role.

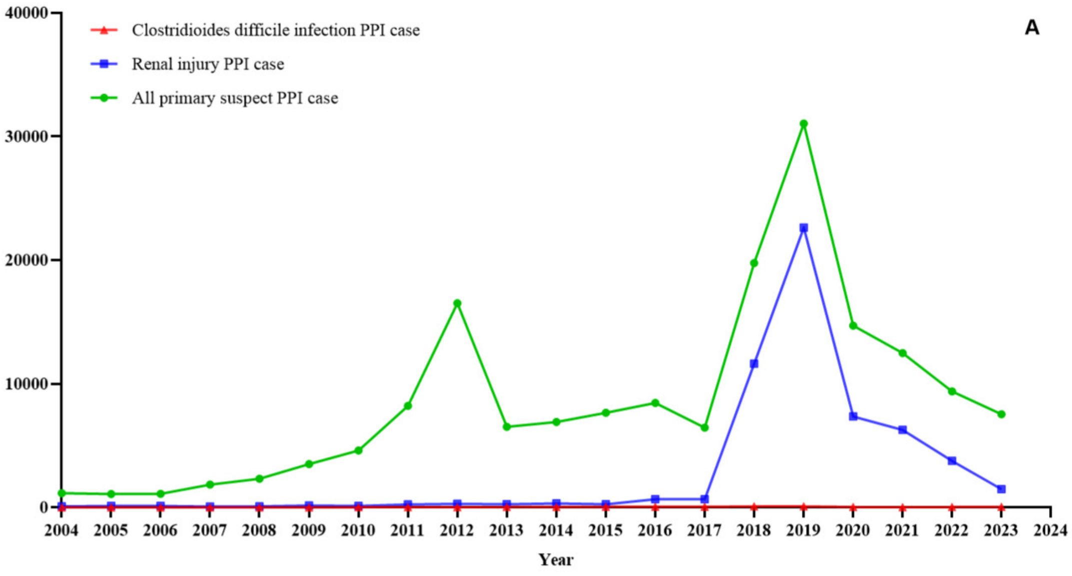


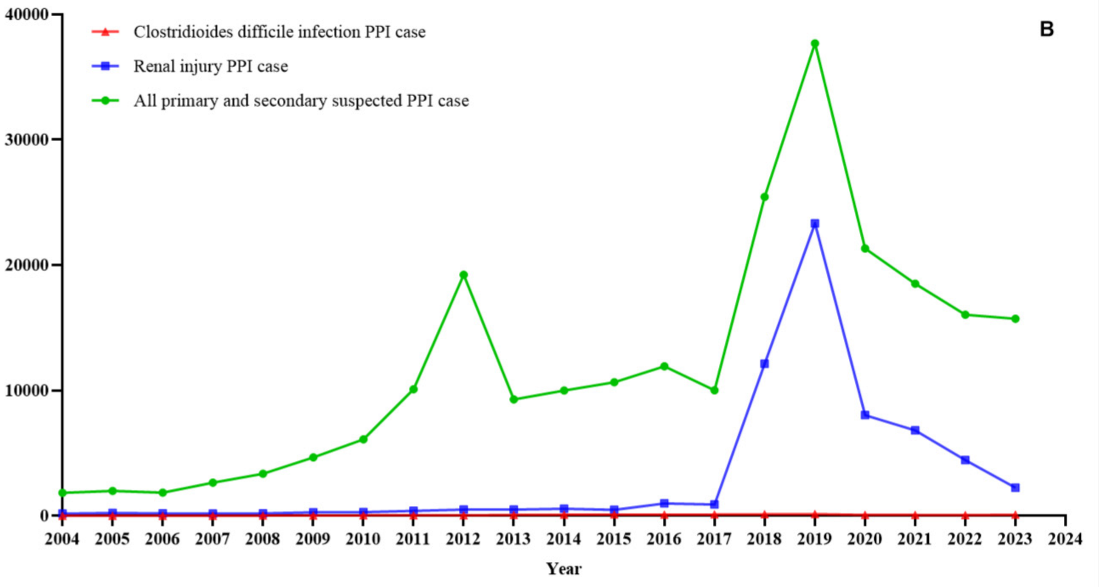

## Supplementary Figure S2

The reported trend of CDI cases associated with PPI treatment from the FAERS database.

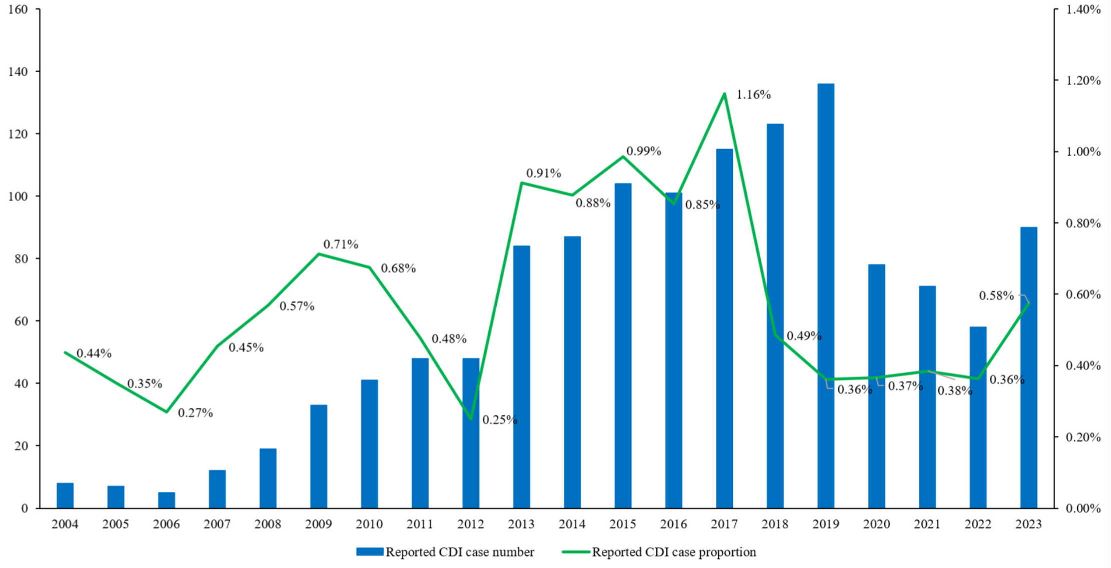

Supplement: Supplementary file 1 [file jcm-15-00230-s001.zip › jcm-4005727-supplementary.pdf]
